# Developing a best practice guide for integrating spiritual care interventions in chronic pain therapy: a qualitative Delphi study

**DOI:** 10.3389/fpain.2025.1682702

**Published:** 2025-11-14

**Authors:** Joël Perrin, Karin Hasenfratz, Simon Peng-Keller, Michael Rufer, Rahel Naef

**Affiliations:** 1 University Hospital Zurich, Zurich, Switzerland; 2 Faculty of Theology and the Study of Religion, University of Zurich, Zurich, Switzerland; 3 Center for Psychiatry and Psychotherapy, Hospital Zugersee, Triaplus AG, Oberwil-Zug, Switzerland; 4 Department of Psychiatry, Psychotherapy, and Psychosomatics, Psychiatric University Hospital Zurich, University of Zurich, Zurich, Switzerland; 5 Institute for Implementation Science in Health Care, Faculty of Medicine, University of Zurich, Zurich, Switzerland; 6 Centre for Clinical Nursing Science, University Hospital Zurich, Zurich, Switzerland

**Keywords:** chronic pain, best practice guide, health professionals, Delphi study, spiritual care, chronic pain patients

## Abstract

Chronic pain patients (CPPs) often face complex, multifactorial challenges, with many reporting that their pain management lacks comprehensiveness. Spiritual care has emerged as a potential resource in addressing the diverse needs of CPPs, but remains underutilized due to healthcare professionals' (HCPs) uncertainty about how to integrate it into clinical practice. This study aimed to develop a best practice guide for integrating spiritual care into chronic pain therapy using a qualitative Delphi study. Three rounds of data collection, involving a panel of CPPs and HCPs with expertise in chronic pain from various disciplines, were conducted. Participants shared their experiences and suggestions for addressing spiritual aspects in pain therapy. The process led to the formulation of a consensus-based best practice guide, outlining practical strategies for HCPs to engage with spiritual care in a way that is respectful and sensitive to individual patient needs. Results indicated that incorporating spiritual care in chronic pain therapy can enhance therapeutic relationships, foster more meaningful patient interactions, and provide additional coping mechanisms. The guide was rated as clinically applicable, and offers a structured yet flexible framework for integrating spiritual care into multimodal pain treatment and is expected to improve patient outcomes by addressing existential aspects of chronic pain.

## Introduction

About 20% of the European population lives with chronic pain (1), which is a multifactorial, complex condition that affects all dimensions of being (2, 3). Up to two thirds of the chronic pain patients (CPPs) consider their pain management to be insufficient (4, 5); many of them do not feel understood by their health care professionals (HCPs) (6). The high symptom burden, which leads to restrictions in everyday life and limitations in meaningful social interaction, coupled with a lack of effective treatment, may raise existential questions of meaning (7). A comprehensive, holistic and person-centered approach to health care, as recommended by the WHO (8), requires HCP to address the spiritual dimension of health (9–13).

Studies have shown that spiritual care is associated with a range of positive health outcomes (14–17), including overall well-being and a constructive interpretation of illness experience (18–20), which are of importance for CPP. In previous studies about CPPs' and HCPs' spiritual needs and concerns it was shown that up to 60% of participating CPPs would like spiritual aspects of health to be included in the treatment process, regardless of their religious believes or denomination (21).

Despite potential benefits, the spiritual dimension of health is seldomly addressed in multi-modal pain treatment (22): Both groups, HCPs and CPPs, seem to perceive spiritual aspects as a very private matter or even a taboo (23). They report to be unaware that the spiritual dimension is an integral part of health care (23). Although HCPs are generally open to integrating the spiritual dimension of health into care, they hesitate due to a lack of best practice guidance, time constraints, and inexperience (23, 24).

To address the lack of guidance in clinical practice, a best practice guide for the integration of spiritual care interventions in chronic pain therapy was developed. The aim of this study was to develop an interprofessional expert consensus on integrating spiritual care interventions in chronic pain therapy.

## Materials and methods

### Design

We used a three-round Delphi study (25, 26) with patient and health professional experts on chronic pain, which was carried out between December 2020 and July 2022. The Delphi method was chosen as it is regarded as a suitable method for research questions that require gathering subjective information from experts (27) in a given area of uncertainty or lack of empirical evidence (25, 26), and where no consensus has been reached before (28). Following Delphi standards (29, 30), the first round used open and broad questions, followed by more specific, in-depth ones in subsequent rounds. The data from each round was analyzed qualitatively and descriptively and was incorporated into the questions for the second and third round (25, 31).

### Setting and participants

Participants (*n* = 47) included CPPs and HCPs (physicians, nurses, social workers, physiotherapists, occupational therapists, psychologists) working with chronic pain patients in the German-speaking part of Switzerland.

Inclusion criteria for HCPs were regular clinical activity with CPPs for at least one year in acute, rehabilitation, community or primary care setting, an age of at least 18 years and no research activity in the area of spiritual care in order to exclude specific in-depth expert knowledge in this area as far as possible. Students were excluded.

Inclusion criteria for CPPs was a diagnosis of chronic pain [pain for ≥6 months with a pain intensity of ≥5 on the 11-point Numeric Rating Scale (NRS; 0 = no SZ, 10 = worst pain)] due to non-terminal illness.

### Data collection processes

Experts were selected by the research group following the above criteria. Recommendations by selected experts were used to recruit further experts (snowball sampling), still according to the defined selection criteria. The same recruitment process was used for round two in order to compensate partially for declining response rates. Since round three was mainly used to evaluate a synthesis of the knowledge generated in rounds one and two, experts who missed round one and round two were excluded from round three.

Each expert was assigned an individual and group-specific code for pseudonymization. Communication with the experts was effectuated via email for the entire study to minimize group pressure for conformity (32), dominant voices taking over the discussion (31), or the influence of seemingly deemed superior expert individuals over others (33).

The first Delphi round was conducted in December 2020, the second in September 2021, the third in June 2022. For each round, the experts had a response time of two months, with an additional month after one reminder, sent out per e-mail. The data collection form with questions for each of the three rounds was accompanied by a cover letter and further documents including the most recent version of the best practice guide in German, all of which can be found in Supplementary Material. The final version of the best practice guide and other resources are available for download at https://www.spiritualcare-leitfaden.ch.

#### First Delphi round—drafting the best practice guide

The goal of the first round was to map and explore which spiritual care aspects were seen as most important by the experts to integrate into multimodal pain management. The questions (Table 1) were deliberately open and based on findings from former studies performed by the research team (21, 23, 24, 34). A definition of *spirituality* that is established in the health care context was introduced at the beginning to ensure a common departure point (35). A multidimensional understanding of *spiritual distress* (36) and *spiritual well-being* (37) served as the theoretical basis for this study. We intentionally chose a broad and inclusive definition of *spirituality* as a dynamic dimension (38) or a *travelling concept* (39), persons with no religious affiliations are not excluded (40) and different backgrounds are acknowledged (41).

**Table 1:** Questions used in the three Delphi rounds.

Questions used in the three Delphi rounds	
Question No.	First Delphi round—introductory questions
1	From your experience, what could be suitable starting points for addressing spiritual aspects (both resources and burdens)? How can these be recognized?
2	Are there questions, phrases, images, or metaphors that have worked well in conversations with patients about spiritual resources and stresses?
3	What do you see as key factors that make it difficult to address the spiritual dimension in treatment?
4	Suppose your patients were to fill out the assessment instrument we developed (see appendix) as part of their treatment. How would you address the answers in the treatment? What would you focus on and how?
Question No.	Second Delphi round—follow-up questions
1	Does the guide in this form reflect your suggestions from the first round? Do you have any additions or suggestions for adaptation?
2	How do you rate the appropriatness of the guide for clinical work with persons with chronic pain (comments on content are also very welcome)? Please give an assessment using NRS 1 (=absolutely unusable)—10 (=perfectly suitable) and a brief written explanation.
3	Metaphors that are experienced as helpful for the conversation have been mentioned repeatedly: (a) Do you have good experiences with certain metaphors? With which ones? (b) Would picture cards/photos help? If so, in what form/which pictures?
4	What further support/additional materials could be helpful for such clinician-patient interactions?
5	How important do you think it is to document the explored r/s resources and burdens in the patient dossier or similar and what should be paid attention to?
6	What further assistance would you need or recommend for the implementation of the guide? What collaboration would you recommend?
Question No.	Third Delphi round—follow-up questions
1	What experiences have you had with the guide? Do you have any input on adjustments to the content or design?
2	With (approximately) how many patients (nominally and/or as a percentage) have you used the guide or individual questions from it in recent weeks?
3	How suitable is the guideline in clinical practice? Please give an assessment using NRS 1 (=absolutely useless)—10 (=perfectly suitable).
4	Does the guide in this form reflect your suggestions from the second round?

#### Second Delphi round—refining the best practice guide

The questionnaire used in the second round (Table 1) included a mix of six open and close-ended questions that were formulated based on the findings from the first Delphi round. In addition, the experts were asked to evaluate a first digital version of the best practice guide, which the research team had drafted based on the results of the first Delphi round. In particular, the experts were asked whether they considered the first version of the best practice guide appropriate and helpful in a clinical setting. The experts' feedback on suitability of the best practice guide was elicited narratively and with a numerical rating scale (NRS).

#### Third Delphi round—implementing the best practice guide

Before the third and final round, the best practice guide was further refined, graphically redesigned and adapted according to the findings of the second round. The most significant adaptation was the separation of the best practice guide into a pocket card and an accompanying booklet, according to the experts input (see *Results*). The final version of the best practice guide, along with five follow-up questions, was distributed to the experts for review (Table 1). This time, the experts were asked to use the best practice guide in practice and then report on their experiences by evaluating the clinical applicability, again both in narrative form and by using a numerical rating scale (NRS).

### Data analysis

After each Delphi round, expert answers were collected, pseudonymised, and analyzed using qualitative content analysis according to Mayring (42, 43), which was supported by using HyperResearch®, a computer-aided qualitative data analysis software for semantic coding. The content analysis method aims to maintain a source's complexity while systematically condensing its content and sort it into categories. It does so by capturing the content of the source text using *in vivo* codes before grouping them, therefore increasing confirmability and traceability of the results (44).

In each round, the codes and categories were generated abductively (45, 46), which means that they were formed both deductively, top-down starting from the research question, and inductively, driven bottom-up by the data and the codes. During this process, each code and category were described and defined in a standardised format to maintain intersubjective neutrality. Codes were justified by a comment if it could not unambiguously be assigned to a text passage. Text passages could fall under different codes, if they fulfilled the respective code criteria. An example of the coding procedure is given in Table 2.

**Table 2:** Example for the coding procedure.

Category	Conversation_content
Subcategory	Steer conversation toward spirituality via inquiry about potential stresses
Code	Addressing stress enables transition to spiritual aspects
Code description	Expert uses the stress discussed as a transition to spiritual aspects. In contrast to “Addressing the intrapersonal coping strategy of the CPP enables transition to spiritual aspects”, the focus is on a subjectively perceived burden for the patient, less on a possibly counterproductive coping strategy.
Examples	1_107_02: […] “When you think about the back pain is there anything that is bothering you as a whole person or that is good for you as a whole person?” 1_504_04: […] In the case of conspicuous spiritual burdens, I would bring this up and ask whether the person concerned has already talked about it with someone else […].

The first Delphi round resulted in a set of 45 different codes that were grouped into 13 categories, some of them including subcategories. The second round resulted in 34 codes grouped into 7 categories, and the third round yielded 11 codes in 3 categories. Numerical rating data were expressed as median and range.

### Rigour

Following the standards of qualitative-explorative research, the trustworthiness (47) of the analytic results after each round was fostered through critical discussions between the first and second author, both with experience in qualitative-explorative research, the Delphi method and the data analysis used. Before each round, the result of the analysis as well as the set of expert questions were presented and agreed upon by all the members of the research group.

## Results

### Participants

A total of 44 HCPs and 3 CPPs participated. The majority of HCPs worked in acute medical and outpatient settings with a few working in rehabilitative institutions. While the response rate of the experts contacted across the three rounds declined, the diversity of the responding expert group remained relatively constant, with CCPs having the highest response rate throughout (Table 3).

**Table 3:** Overview of participants and response rates.

Profession (Code)	First round: contacted/responding	Second round: contacted/responding	Third round: contacted/responding
Physicians (1xx)	19/8	20/8	10/3
Nurses (2xx)	4/2	4/2	3/0
Social workers (3xx)	2/2	2/0	2/0
Physiotherapists (4xx)	7/4	7/3	3/0
Occupational Therapists (5xx)	4/4	4/2	4/1
Psychologists (7xx)	2/1	2/1	3/1
CPPs (6xx)	3/3	3/3	3/2
Total	41/24 (56%)	42/19 (45%)	28/7 (25%)

### Overview of results across the three Delphi rounds

Analysis of the first Delphi round showed that HCP-CPP communication around spirituality can be structured into three overlapping phases with specific themes (Table 4), namely:

Phase *a) Creating context for interaction* before and while initiating the conversation; further differentiable into *a_1_) Acknowledging challenges* and *a_2_) Setting the stage for meaningful conversations.*
Phase *b) Starting intentionally* the phase of topic progression and exploration; further differentiable into *b_1_) Initiating the talk* and *b_2_) Exploring topics for intervention*.
Phase *c) Concluding the interaction purposefully*; further differentiable into *c_1_) Explicating as an intervention* and *c_2_) Considering additional interventions*.

**Table 4:** Results—overview.

Phase	Themes	Conversations about the spiritual dimension of health in a therapeutic setting …
a) Creating context for interaction	a_1_) Acknowledging challenges	… are considered challenging by HCPs for a wide variety of individual, societal, and institutional reasons.
	a_2_) Setting the stage for meaningful conversations	… are more likely to occur given sufficient time availability, a private setting, and openness of the HCP to the topic.
b) Starting intentionally	b_1_) Initiating the talk	… can be initiated by HCPs both directly and indirectly. Metaphors can support the transition to and conduction of conversations on spiritual aspects.
	b_2_) Exploring topics for intervention	… can best be initiated by HCPs through: Exploration of resources Understanding CPP's metaphors or by using metaphors oneself as a HCP Addressing concepts of illness
c) Concluding the interaction purposefully	c_1_) Explicating as an intervention	… can be seen as an intervention in itself, in the form of an investment in the therapeutic relationship. The conversation should be documented to improve current and future treatment.
	c_2_) Considering additional interventions	… can give way to specific interventions such as: Exploring and strengthening spiritual resources or integrating them into individual treatment goals Searching for new resources Developing a joint concept of illness Consolidating or resolving metaphors together with the CPP

These phases provide insight into how clinicians can better address spiritual care during interactions with chronic pain patients, and were used to structure the best practice guide.

The findings from the second round were used to adapt and refine the results of the first round by further specifying the key topics of the best practice guide, namely Phases b) and c): These two phases are strongly related to the HCP-CPP communication itself and can easily be influenced by individual HCPs, whereas Phase a) refers to the context in which the communication takes place and requires organizational measures.

After drafting and refining the guide in the first two rounds, the third round of the Delphi study focused on evaluating its clinical applicability. This final evaluation provided key insights into how the guide could be practically implemented in various clinical settings, and further informed the findings structure, resulting in six key themes informing the guide (Table 4):

### First Delphi round—drafting the best practice guide

#### Phase a) Creating context for interaction

Conversations about spiritual concerns often come with uncertainty and unique challenges, requiring a supportive setup. Openness, trust, and understanding were reported to be important prerequisites for making room for spiritual issues to be addressed. Anchor examples supporting the results can be found in Table 5.

**Table 5:** First Delphi round—anchor examples.

First Delphi round—anchor examples		
Theme	Code	Anchor example
Phase a) Creating context for interaction		
a1) Acknowledging challenges	Expectations make it difficult to address the spiritual dimension	404_03: I am a physiotherapist. Expectation and order from patients, employer, health insurance that I mainly take care of the knee, shoulder, etc.
	Aspect of boundary violation makes it difficult to address the spiritual dimension	105_03: it is much easier to ask the patient about his sexuality than about his spirituality. This topic is at least to a certain extent occupied by shame, viewed on a meta-level, of course, completely unjustifiably.
	Lack of thematic reflexion makes it difficult to address the spiritual dimension	601_01: Personally, I think that in the case of a spiritual question or problem, I could also provide too little assistance and therefore would not bring it up on my own.
	HCP has no specific approaches in conversations on spiritual aspects	202_02: Experience and literature show that professionals often lack words.
	Lack of time makes it difficult to address the spiritual dimension	601_05d: Due to limited time and also lack of training, I did not address the importance of daily yoga practice.
	Somatic issues make it difficult to grasp the spiritual dimension	101_03: Pronounced pain symptoms that dominate the patient's entire experience, leaving no room for the topic of “spirituality”.
a_2_) Setting the stage for meaningful conversations	Conversation on spiritual aspects requires openness	105_01: There are also always patients who talk about their spirituality between the lines or quite openly.Then “the thread” can be picked up well. 501_02: In my experience, patients do not bring spiritual thoughts into therapy on their own and this happens even less the more technical the wording and instructions are. For example, when someone is told to sit up, “Try to straighten your chest”. sounds different than “Lift your heart against the ceiling”.
	Conversation on spiritual aspects requires understanding	106_03: [Spirituality eventually] is also too personal, they don't want to give away too much of their inner self, knowing that the other person's understanding is not necessarily given.
	Conversation on spiritual aspects requires trust	502_03: The prerequisite is always a trusting relationship with the patient. 601_03: I think that it is unfamiliar for both the practitioners and the patients to talk about spiritual concerns, because it is a very personal topic that is associated with a lot of shyness. So there is probably a lot of uncertainty on both sides.
Phase b) Starting intentionally		
b_1_) Initiating the conversation	Patient expresses a desire to talk about spiritual aspects	105_01: There are also always patients who talk about their spirituality between the lines or quite openly.
	Patient directly expresses wanting to talk about spiritual aspects	105_01: There are also always patients who talk about their spirituality between the lines or quite openly.
	Spiritual aspects are asked indirectly	402_01: I am rather reluctant to address them directly. Most of my patients were born abroad. Thus, in most cases, their spiritual roots can hardly be assessed. During the exploration of resources and positive/healthy behavioral strategies, spiritual aspects are often addressed. Mostly these are assessed as resources recognized.
	Spiritual aspects are asked directly	104_02: “Are there powerful, helpful and strength-giving transcendent inner images that help them in crisis situations?”
b_2_) Exploring topics for intervention	Addressing resources enables transition to spiritual aspects	401_01: In the conversation, look at the various resources and from these resources come into the conversation about possible spiritual support. 603_04: And in general I would come about resources and what gives energy, longings, dreams etc. on deficiently perceived aspects instead of directly about burdens.
	Addressing situations that make the pain more bearable allow transition to spiritual aspects	107_01: “Are there moments, places, or encounters that you feel make the pain more bearable?”
	Addressing CPPs intrapersonal coping strategy enables transition to spiritual aspects	101_04: Would I address basic life attitudes and clarify what “rails” he wants to lay or has already laid for his life. 107_02: “When you think about the back pain is there anything that is bothering you as a whole person or that is good for you as a whole person?”
	Addressing meaningfulness allows for transition to spiritual aspects	104_01: Question about inner images experienced as meaningful and comforting, previous access to religiosity or lived spirituality. 102_02: “What gives meaning to your life? what still keeps you alive?, what gives you strength?”
	Addressing thoughts that make the pain more bearable allows transition to spiritual aspects	503_02: Very different: Sometimes we use the image of the car that also needs gasoline to drive.Thus the question: Where do you refuel? Or what is the gasoline for them? 301_02: Positive formulations and images from the pain-free phase with the focus of the coping strategy through the inclusion of spiritual aspects are extracted.
	Addressing concepts and explanatory models enables transition to spiritual aspects	104_06a: CPP with migrant background from Serbia experiences her severe work accident and the subsequent psychosocial consequences as divine punishment. 107_01: Letting the pain tell: for example, “Are there moments, places, or encounters that you feel make the pain more bearable? What do you think makes the pain more bearable?”
	Addressing stresses enables transition to spritual aspects	403_02: Always having to fight and experiencing setbacks—trying to run up a sand dune and not getting off the ground. Frustration of the situation—the grass does not grow faster when you pull on it. Hopelessness and aimlessness—standing at the railroad track but no train comes.
Phase c) Concluding the interaction purposefully		
c_1_) Explicating as an intervention	Intervention already happens by listening	301_06b: Relief can come with just thematizing, as these are very deep-seated and often shame-ridden feelings. 107_06b: Patients often already feel understood when they notice that the medical professional is interested in the individual explanation pattern in the sense of transcultural sensitivity.
	Intervention happens through interdisciplinary collaboration	402_06b: In the case of spiritual stresses, I offer space for the patient's narrative. In doing so, I make sure that what is said is manageable for me. If the patient brings up deep-seated spiritual injury, I try to mark the boundaries of physiotherapy. In the best case, I succeed in pointing out to the patient that the content is a good topic for psychotherapy.
	Spiritual aspects are addressed repeatedly	201_01: It seems important to me that the spiritual aspects are not asked once, but are taken up and addressed again and again in the course. 202_06c: Take your time, also say that you have time. Express that you can understand [the patient]. Bring it up in several conversations … in stages.
c_2_) Considering additional interventions	Intervention by addressing the spiritual dimension as part of the therapy goals	108_05b: Nature as a “place of power” was used to achieve a concrete treatment goal (to be able to go to the place of power again). For the patient, spirituality was the motivator, so to speak. 106_05d: Physical therapy alone would not have motivated the patient, but the prospect of being able to go back into the forest would have.
	Intervention through direct promotion of circumstances that make the pain more bearable.	102_07b: With the patient, try to find the really important things in life, to live in the here and now. What is really important.
	Intervention through exploration of alternative resources	402_05c: I try to find out why the place of power (e.g., forest, home, or mountains) is important and what the place triggers in the person. The focus is on the physical reactions. Then we try to explore whether this positive feeling can be reproduced in other places or other activities.
	Intervention by promoting access to existing resources	107_05b: To talk primarily about the forest, the vacations or the Jasskränzchen [social Swiss card game] and in this way, without making direct recommendations, to stimulate thoughts.
	Intervention by dissolving negative concepts	106_06b: Correct an idea that is burdensome for the patient, where the disease comes from 102_06b: Clarify the question of guilt, Often people feel guilty where they are not guilty but did not fulfill the expectations of others.
	Intervention by reversing a negative connotation	107_05a: I often had similar experiences: It seems important to me that the negative connotation is transformed into a positive one: Instead of “I can't do all that anymore” better “if I could, it would certainly do me good”. This can be about places like in the example “forest”, about points in time like “on vacation” or also about human encounters “in the Jasskränzchen”.

##### Theme a_1_) acknowledging challenges

HCPs described conversations about spiritual aspects with CPPs as particularly challenging. On the one hand, general reasons were mentioned, such as an overwhelming pain symptomatology, different socio-cultural backgrounds, or language barriers, all three of which make conversations between HCP and CPP complex and often difficult. HCPs also mentioned their own and society's approach to and significance of spirituality as a difficulty for a successful start to the conversation. For HCPs, addressing the spiritual dimension, which was often a taboo topic in the clinical setting, was sometimes associated with a feeling of transgressing boundaries. A majority of the experts expressed uncertainty: They stated that they lack training and personal familiarity with spiritual care as well as a conversational repertoire including vocabulary, suitable questions, expressions, images or metaphors for a conversation about spiritual aspects in chronic pain. HCPs experienced the role of spiritual care in chronic pain therapy to be intertwined with potentially divergent and interplaying expectations of patients, the health system, and society at large. Lastly, a large number of experts also described a lack of time resources as an obstacle.

##### Theme a_2_) setting the stage for meaningful conversations

Spiritual aspects were often understood by the experts as a very private topic. It was important for them to set the stage in a way that fosters meaningful conversations—whether the conversation is planned or arises spontaneously. Almost all experts stated that a conversation about spiritual aspects requires an authentic and interested attitude toward the topic. HCP's own examination of and reflection on spirituality helps to be open and curious about the CPP's perceptions, even if it may differ from their own stance. It was acknowledged that spiritual aspects are not equally important for all patients, but there was consensus that all those who have a need to talk about them are more likely to do so if the other person signals openness and availability. The experts repetitively pointed out that especially in the often very busy inpatient setting, it is important to create privacy for conversations about spiritual aspects in order to open up a space in which personal views and experiences could be shared. If a conversation on spiritual topics is planned, it is worth reserving sufficient time for it, alike it is already practice for conversations on other topics considered intimate.

#### Phase b) starting intentionally

HCPs mentioned various topics that can serve as an introduction to a conversation about spiritual aspects with CPPs by either addressing them directly or indirectly. Prominently and frequently mentioned topics are the exploration of spiritual resources, the recognition or use of metaphors or symbolic language as well as the discussion of illness understandings and burden of illness. Anchor examples supporting the results can be found in Table 5.

##### Theme b_1_) initiating the conversation

Examples in the data showed that the initiative for conversations about spiritual aspects was taken more often by the HCPs than by the CPPs. This is why HCPs stressed the need for an openness and familiarity with spirituality as an essential part of therapeutic interactions in health care. The experts felt that conversation about spiritual aspects with CPPs could be initiated in direct and indirect ways, with them having no general preferences. They argued for an indirect entry, e.g., by addressing patients’ resources in a general way if it is unclear what significance spiritual aspects have for the CPP. Indirect approaches therefore may not lead to a spiritual topic, but to other areas that are important for the therapy of CPP. Depending on the context and the care process, spiritual aspects may be approached again later. According to the experts, specific spiritual resources or distress can also be asked about directly if the context, relationship, and previous discussions allow for it.

##### Theme b_2_) exploring topics for intervention

A clear majority of the experts reported that an exploration of resources, which is reflective of an indirect approach, can be a useful starting point to address spiritual aspects. Examples include questions about what helps or helped the CPP to bear the pain in the sense of intrapersonal coping strategies; addressing concrete situations that made the pain bearable or addressing a search for meaning caused by the chronic pain. Another way to start addressing spiritual aspects is by using symbolic language or by asking for CPP's metaphors for chronic pain. The experts repeatedly stated that metaphors, proverbs, and idioms expressed by the CPP may be indicative of their beliefs. Use of metaphors was considered particularly helpful as some CPPs are better at communicating in images than abstract words. According to the experts, these images can be taken up or developed together with the CPP.

#### Phase c) concluding the interaction purposefully

Most experts viewed conversations on spiritual aspects as an accessible, generalist intervention in itself. In addition, various starting points for further specific spiritual care interventions emerged from the experts' responses. Anchor examples supporting the results can be found in Table 5.

##### Theme c_1_) explicating as intervention

Even the low-threshold inclusion of spiritual aspects into interactions with CCPs was understood as an investment in the therapeutic relationship and reflective of spiritual care, which may mean that additional, specialized interventions may not always be necessary. So, according to the experts, an open conversation that does not exclude spiritual aspects can be understood as a spiritual intervention because it can trigger CPP's reflection on spirituality and its potential for their health and well-being. Moreover, many experts emphasized the importance of interdisciplinary collaboration, which is necessary for conversations, especially about spiritual aspects.

##### Theme c_2_) considering additional interventions

Sometimes specific interventions relating to spiritual aspects were also considered useful by the experts. Possible interventions mentioned by the experts were, for example, exploring and strengthening spiritual resources or integrating them into individual treatment goals, opening up new resources, or addressing negative concepts of illness that lead to spiritual distress.

So, according to the experts, the spiritual dimension can be taken into account in jointly formulated therapy goals. This can be done in the sense of promoting access to spiritual resources or integrating them into therapeutic actions, e.g., through targeted occupational or physical therapy, pain therapy or everyday life planning. If, for example, a CPP had experienced long walks in the woods as spiritually healing before becoming unable to walk longer distances, physiotherapy could enable the CPP to do so again.

Similarly, the patient can be encouraged to search for alternative and new resources. Chronic pain or illness and the associated limitations potentially make it difficult or impossible to access existing spiritual resources. According to the experts, the point here is not to make suggestions to the patient or even to impose something on the CPP. What is meant is the accompaniment of a search for new supportive values, experiences or practices. Initially, this sometimes involves simply acknowledging the losses experienced.

The explanation of the origin of pain and the joint development of an individual illness understanding is an important basis in pain therapy. If, according to the experts, it becomes clear that spiritual distress (such as feeling punished by God) are related to the illness, addressing them can be important for the patient. CPPs often find it helpful and relieving to be able to talk about and understand this spiritual distress. An ongoing accompaniment and support in developing a less stressful perspective can be important for the therapy. For a low-threshold access to all specific interventions, a search for and unfolding of helpful metaphors is again seen appropriate. This is often implicitly recognized by the experts when they repeatedly speak figuratively of opening up “places of power”.

### Second Delphi round—refining the best practice guide

#### Phase b) starting intentionally

In the second Delphi round, expert feedback pertained mainly to *b) Starting intentionally* and *c) Concluding the interaction purposefully*. The focus was especially on how to structure conversations around spiritual aspects by using specific techniques, such as images and language. Anchor examples supporting the results can be found in Table 6.

**Table 6:** Second delphi round—anchor examples.

Second Delphi round—anchor examples		
Theme	Code	Anchor example
Phase b) Starting intentionally		
b_2_) Exploring topics for intervention	Expert works with metaphors	401_03a: Images of nature such as trees, rivers, landscapes contribute a lot to stability; more often I have also experienced images of movement as power images, such as sailing, mountain climbing, etc.
	Expert expresses positive opinion about metaphors	111_03a: Yes, vivid comparisons help to convey content, often helpful when related to the patient's world of experience. 503_03a: Yes! However, rarely in an initial interview. Images are more likely to emerge in the work with clients. Perhaps also to change beliefs, to develop new power images.
	Expert expresses positive opinion about physical images	105_04: Personally, I always like to work with drawings, mind maps, etc., but these are developed together with the patient on a sheet of paper or on a flip chart. 110_03b: Could be supportive via the additional visual input.Would also be an option with people from immigrant backgrounds. 601_03b: For people who may find it more difficult to have an abstract conversation or who may not understand everything due to a language barrier, picture cards might be helpful. The picture of the sand dune and the garden are very helpful for me.
	Expert expresses herself ambivalently to negatively about picture cards/photos	201_03b: Rather impractical or costly due to hygiene regulations.
	Expert suggests physical objects with image meaning	101_04: Instead of cards, one could use objects with analog content, or have the patient bring them: Object that relieves pain, strengthens the patient. 701_04: Possibly natural materials (e.g., stones, flowers, roots,…) or symbolic objects (e.g., wooden figures, candles).
Phase c) Concluding the interaction purposefully		
c_1_) Explicating as an intervention	Documentation is important	102_05:Be sure to write down resources or techniques that help deal with acute pain. So that one can remind the patients in the given case again. 112_05: Importance of religion/spirituality should be given the same weight as family or work. 203_05:Possibly brief documentation, possibly create patient documentation in Kisim [common clinical information system in Switzerland] on request, whether desired and permitted by the patient. Explain that in principle it is also important for the entire treatment team to know what resources are available or whether beliefs are present. But actually only in terms of “what is important for the treatment team” document from my point of view.
	Freedom from judgment and respect are important in documentation	101_05: This seems very important to me, because this is relevant information for all those treating. However, it is important to document r/s aspects respectfully and not in a judgmental way. Documenting also lifts spirituality out of the taboo sphere, especially if the patient notices or is told about it. 110_05: Documentation is important, also with regard to possible changes of therapists, to check agreed goals, etc; but also in the sense of multimodal pain therapy. In the latter context, coordinated collaboration of the various disciplines under the same concept with the same goal is crucial. Care must be taken to ensure that documentation is free of judgement
	Documentation should be discreet	105_05: I could imagine that the topic of religion and spirituality is even more shameful than questions about sexuality. For this reason I would recommend to make only very superficial documentations concerning this. Such as: has spiritual needs or is open about spirituality. Nothing more.
	Spirituality is a taboo subject	603_05: Important but only in consultation with the patient.
c_2_) Considering additional interventions	Example for negative images	101_03a: I find pictures very helpful, especially when they come from the patient. Painting therapy is a valuable approach here. I have often continued to work with images from painting therapy. The fire that burns, the hammer that crushes. Also r/s symbols can appear here spontaneously: Light, darkness, the cross, the hand of God.
	Example for positive images	602_03a: Pictures from nature (tree, meadow, river) 501_03a:—sth. is “in flow”, it is “running” vs. blocked, stagnant—walk, path, steps, vs. seeing a mountain, abyss, obstacle—view, looking back on a way vs. “turning in the wheel”, lack of perspective—creating islands as a symbol for breaks/relief/relaxation

##### b_2_) Exploring topics for intervention

The experts saw value in the use of metaphors to initiate and then focus on spiritual aspects during interactions. Around half of the experts explicitly stated that they had already used them consciously or unconsciously. As in the first round, the experts emphasized the benefits of both physical images and language images in case of cultural or linguistic communication barriers. The experts also repeatedly stressed the importance of taking the physical images of metaphors of CPPs as a starting point to explore them jointly.

HCPs saw value in the use of hardcopy images or objects (such as pictures or puppets), although only a few had experience using them. There were reservations about their use for hygienic reasons.

#### Phase c) concluding the interaction purposefully

In this second round, two topics were relevant, namely how to document spiritual care interventions in *c_1_) Explicating as intervention* and the types of images used for spiritual care intervention in *c_2_) Considering additional interventions.*

##### c_1_) Explicating as intervention

In the second round, almost all experts explicitly stated that it is important to document the content of discussions on spiritual aspects with CPP, e.g., in the patient dossier. Two main reasons were given by the experts: On the one hand, recording spiritual aspects including spiritual resources and distress allows HCP and CPP to jointly set and review goals in multimodal pain therapy. On the other hand, information about the conversations can reach all current and future treatment providers, which is relevant for interdisciplinary collaboration in multimodal pain management. Furthermore, according to the experts, the recording of spiritual aspects in a patient dossier or elsewhere gives them greater weight and visibility. Some experts argued in favor of sketchy documentation only, since extensive documentation could be perceived as intrusive by the CPP. What seemed most important was to maintain prudence in documentation, as should be the case for all potentially sensitive topics in trusting therapeutic relationships.

##### c_2_) Considering additional interventions

Asked about their experiences with suitable examples of metaphors, the experts stated the usage of images with positive connotations significantly more frequently. This applies both to images for moving a conversation towards spiritual dimensions or continuing to work with an image in a therapeutic way. The most frequently mentioned images were those of nature: A tree as a symbol of growth, weathering a storm, home and shelter for birds, a river as a symbol of serenity, continuity or transience. Images of nature often overlap with images of movement and dance, which were also often mentioned: A mountain to be climbed or a garden where one can dance. A third group, repeatedly mentioned, included images from mechanics: A car that needs to be refueled, or a train station at which one waits. Religious symbols, such as a cross or a prayer chain, were hardly used by the experts.

#### Rating of the best practice guide by the expert group

With regard to the suitability of the best practice guide to integrate spiritual care into their clinical work with CPP, the experts gave the refined draft as used in the second round a median NRS score of 8 out of 10 (*n* = 13, min = 7, max = 10). The majority of experts reported narratively that the current version integrated their own inputs from the first round well. The practically oriented structure with examples was appraised to be a clear, helpful and beginner-friendly guide to enable discussion about the spiritual dimension of chronic pain and its management with CPP.

Nonetheless, minor modifications were considered necessary for the guide to be usable in everyday clinical practice for each group of therapeutic professionals, as it would otherwise be too generalizing. Also, some experts wished for an introduction or training in the use of the best practice guide.

There was somewhat conflicting feedback on the desired length and level of detail: Some experts wanted the best practice guide to be more detailed, with more examples and a theoretical background. Other experts noted that it is precisely the brevity and clarity of the current version that make the guide usable in everyday clinical practice—and would even prefer a slimmer, more reduced version.

### Third Delphi round—implementing the best practice guide

The last round focused on rating the clinical applicability of the finalized best practice guide, which was rated with a median NRS score 8.75 out of 10 (*n* = 7, min = 8, max = 10).

Those experts participating in the third round felt that their feedback and expertise that they had provided were well represented in the final version of the best practice guide. The experts praised the inclusion and openness of the guide's examples regarding different forms of spiritual aspects and described it as helpful support for less experienced HCP. Some experts even reported using the best practice guide with success with non-chronic pain patients. As expressed earlier, HCPs saw a need to increase awareness and to offer education and training on how to integrate spiritual care into chronic pain management, based on the best practice guide.

## Discussion

The aim of this study was to develop a comprehensive best practice guide for the integration of spiritual care interventions into chronic pain therapy. To accomplish this, we used a three-round Delphi method involving an expert panel consisting of health professionals and patients with chronic pain. Through iterative rounds and using qualitative methods, we gathered insights on essential components of spiritual care and identified specific challenges in incorporating spiritual conversations in the clinical setting. Each Delphi round allowed for refinement of the guide, aligning expert input with the practical needs of chronic pain management. The guide was finalized based on consensus feedback on its clinical applicability, which was rated as high. It provides a structured framework that facilitates respectful and meaningful integration of spiritual care.

The findings underscore the need for spiritual care as a critical component in multimodal pain management. Experts reported that the guide's structured approach supports HCPs in addressing spiritual aspects with CPPs, enhancing therapeutic rapport, and reducing the taboo surrounding spirituality in healthcare. The resulting guide offers accessible tools, such as metaphors and specific conversation strategies, to foster open discussions about spiritual resources and distress. Overall, the integration of spiritual care in chronic pain therapy, as advocated in the guide, promises improved patient outcomes through more holistic, patient-centered care.

As far as the applicability is concerned, the best practice guide has been found valuable by the experts to provide HCPs with an accessible and usable tool to initiate conversations with CPPs on spiritual resources and distress relevant to their chronic pain management. In daily practice, this can contribute to (a) reducing the taboo surrounding spiritual aspects, (b) addressing them, (c) integrating them into clinical treatment, and (d) documenting them systematically.

a) **Reducing the taboo surrounding spiritual aspects:** As long as spirituality is considered something private or a taboo topic even by experts on chronic pain, the hurdle of addressing it in clinical setting is enormous for less experienced HCPs (48–50). This uncertainty in regard to the inclusion of spiritual care may lead HCPs to be less open regarding spiritual aspects due to the fear of making mistakes or to transgress a patient's sense of privacy. Qualitative findings, which synthesize expert consensus, demonstrate that there are several practicable and feasible ways to address spiritual aspects in conversation with CPPs, especially in light of an inclusive definition of spirituality that is not limited by religious beliefs or faith traditions.
b) **Addressing spiritual aspects:** The experts particularly emphasize the relevance of using metaphors to facilitate provision of spiritual care across interactions and conversations with CPPs (*creating context for interaction, starting intentionally, concluding the interaction purposefully*). Images can be incorporated or picked up at a low threshold in the conversation and facilitate communication across language or cultural boundaries that may exist between provider and patient. In the best practice guide, we included multiple examples for metaphors or questions, as recommended by the experts, together with additional background information in the accompanying booklet.
c) **Integrating spiritual aspects in clinical treatment:** As shown in our previous studies (23, 24), HCPs find it challenging to integrate the spiritual dimension into chronic pain management, which was confirmed in the present study. There is a fear of imposing oneself and intruding into a very personal realm. We were able to show that HCP's apprehension may be mitigated by proopsing different ways to approach spiritual care, for example by addressing them in indirect ways through engaging in a conversation on general resources and capacities. Moreover, we found that it was important for HCP to understand that an invitation to express spiritual resources or distress constitutes a spiritual care intervention. The best practice guide that details concrete and different approaches to spiritual care is is meant to assist HCPs to work with CPPs in a supportive way and to enable them to draw forth meaningful and helpful practices, values and experiences.
d) **Documenting spiritual aspects:** Qualitative findings also demonstrated the need to document spiritual care in the patient record first, to make the importance of spiritual aspects in chronic pain patients visible, ensuring that patient needs for spiritual care are addressed, and second, to enable collaborative, interprofessional spiritual care.

### Strengths and limitations

This study contributes to the growing efforts to promote the integration of spiritual aspects in healthcare. The broad range of experts on chronic pain from different professional backgrounds allowed to incorporate a variety of viewpoints, which is key in Delphi studies. In line with good practices related to Delphi research, the coding system was generated in several deductive and inductive processes. Qualitative content analysis with documentation principles enabled a critical examination of results to be rooted in the original data. Frequent discussions with the entire research group supported the trustworthiness of the results.

Limitations of the study include the rather small sample size with a moderate and declining response rate. The overrepresentation of the expert group of physicians also leads to a bias in favor of their beliefs and experiences, while the voices of the CPPs were relatively underrepresented by the small expert group in comparison to the sum of the different HCP subgroups as a whole.

These aspects underline the necessity of a validation of the best practice guide on a larger scale in follow-up projects. Furthermore, the sampling procedure might have resulted in a selection bias with an overrepresentation of participants who take a keen interest in spiritual care or who have open attitudes towards spirituality. These limitations need to be taken to account when transferring the results of this study to other contexts.

## Conclusions and future directions

Using the Delphi method, we generated an interprofessional expert consensus on spiritual care provision in chronic pain patients, primarily based on HCP perspectives. The consensus informed the development of a best practice guide that was also appraised for its utility in clinical practice. This represents the first step, as no comparable guideline has been developed and tested in this area to date. In a next step, utility and applicability of the guide needs to be evaluated in larger group of HCPs and especially CPPs.

The best practice guide attempts to address many areas of discussing spiritual aspects that experts consider difficult in interactions between HCPs and CPPs. Nevertheless, such a guidance will not be able to replace, but complement spiritual care training in health care staff. In this regard, further interventions are needed to raise awareness and provide continuing education.

Another aspect repeatedly mentioned by the experts that prevents the integration of spiritual aspects is the time factor—and thus, indirectly, the costs. Therefore, cost-benefit analyses of spiritual care need to be conducted, focusing on whether the training for HCPs and the resulting intervention outweighs its cost in terms of efficiency and effectivity.

## Data Availability

The original contributions presented in the study are included in the article/Supplementary Material, further inquiries can be directed to the corresponding author.
