# Supplementary material for: Developing a best practice guide for integrating spiritual care interventions in chronic pain therapy: a qualitative Delphi study
Source: Front Pain Res (Lausanne). 2025 Nov 14;6:1682702. doi: 10.3389/fpain.2025.1682702 (PMC12660185; doi:10.3389/fpain.2025.1682702)
Supplement: Supplementary file 1 [file Datasheet1.pdf]

Leitfaden zur Integration spiritueller Aspekte in die multimodale Schmerztherapie

15.12.2020

Sehr geehrte Damen und Herren

In einer NFP 74 geförderten Studie zu spirituellen Ressourcen und Belastungen bei chronischen Schmerzpatient*innen gaben über 60% der befragten Patient*innen an, dass sie die Berücksichtigung spiritueller Aspekte im Behandlungsprozess wünschen. Die Schmerz-Fachpersonen gaben wiederum an, diesem Wunsch grundsätzlich positiv gegenüberzustehen, zeigten sich jedoch aufgrund fehlender Modelle, knapper zeitlicher Ressourcen und unzureichender Erfahrung in diesem Bereich zurückhaltend. Gleichzeitig belegt die jüngere Forschung die Wichtigkeit der spirituellen Dimension im Umgang mit chronischen Schmerzen.

Um Fachpersonen ein kurzes Screening-Instrument in die Hand zu geben, haben wir in einem ersten Schritt einen Fragebogen zur niederschwelligen Erfassung spiritueller Ressourcen, Belastungen und Umgangsstrategien (SDRQ) entwickelt und validiert. Wir möchten nun in einem zweiten Schritt einen Leitfaden zu der Frage erarbeiten, wie die spirituelle Dimension im Behandlungsprozess niederschwellig und gewinnbringend in eine multimodale Schmerztherapie integriert werden kann.

Gerne möchten wir Sie als klinische*n Expert*in einladen, an einer Delphi Umfrage teilzunehmen. Das Ziel dieser Umfrage ist es, Ihre Erfahrungen und Einschätzungen zum Thema in die Entwicklung eines entsprechenden Leitfadens einfliessen zu lassen.

Die Delphi Umfrage verläuft in anonymisierter Form in zwei Runden. Wenn Sie teilnehmen möchten, werden Sie in den nächsten Wochen ein Dokument mit acht Fragen per E-Mail erhalten (*erste Runde*). Ihre Rückmeldungen werden wir qualitativ auswerten. Einen auf den Ergebnissen der ersten Umfrage basierenden Vorschlag für einen Leitfaden sowie noch offene Fragen werden wir Ihnen abermals vorlegen (*zweite Runde*). Selbstverständlich werden wir Ihre Mitwirkung auf Wunsch in der geplanten Publikation vermerken.

Wir würden uns sehr freuen, wenn Sie an diesem Prozess mitwirken, und danken Ihnen für Ihre Antwort.

Freundliche Grüsse
Das Studienteam

Prof. Dr. Simon Peng-Keller[1], Prof. Dr. Michael Rufer[2], Prof. Dr. Rahel Naef[3], cand. med. Joël Perrin[1], pract. med. Karin Hasenfratz[1]

---

1 Professur für Spiritual Care, Universität Zürich
2 Zentrum für Soziale Psychiatrie, Klinik für Psychiatrie, Psychotherapie und Psychosomatik, Psychiatrische Universitätsklinik Zürich
3 Zentrum Klinische Pflegewissenschaft, Universitätsspital Zürich & Institut für Implementation Science in Health Care, Universität Zürich
