# Supplementary material for: Developing a best practice guide for integrating spiritual care interventions in chronic pain therapy: a qualitative Delphi study
Source: Front Pain Res (Lausanne). 2025 Nov 14;6:1682702. doi: 10.3389/fpain.2025.1682702 (PMC12660185; doi:10.3389/fpain.2025.1682702)
Supplement: Supplementary file 2 [file Datasheet2.pdf]

FNSNF
FONDS NATIONAL SUISSE
SCHWEIZERISCHER NATIONALFONDS

Universität Zürich[UZH]

74 NRP
Smarter Health Care
National Research Programme

Erste Runde der Delphi-Umfrage: Leitfaden zur Integration spiritueller Aspekte in die multimodale Schmerztherapie

Fragebogen

Angaben zu Ihrer Person

Datum:

Beruf:

Alter:

Setting:

Akutspital

Rehabilitation

ambulante Versorgung

anderes: _ ______________

Fragen zur Integration der spirituellen Aspekte im Behandlungsprozess

1. Was könnten aus Ihrer Erfahrung geeignete Anknüpfungspunkte sein, um spirituelle Aspekte (Ressourcen und Belastungen) anzusprechen? Wie können diese erkannt werden?

2. Gibt es Fragen, Formulierungen, Bilder oder Metaphern, welche sich in Gesprächen mit Patienten über spirituelle Ressourcen und Belastungen bewährt haben?

3. Welches sind für Sie zentrale Faktoren, welche es erschweren, die spirituelle Dimension in der Behandlung[1] aufzugreifen?

---

[1] Mit *Behandlung* sind auch Beratung, Pflege, Therapie, Begleitung, etc. gemeint

4. Angenommen, ihre Patient*innen würden im Rahmen der Behandlung den von uns entwickelten Fragebogen (s. Anhang) ausfüllen. Wie würden Sie die Antworten in der Behandlung[1] aufgreifen? Worauf würden Sie wie fokussieren?

Im folgenden lesen Sie drei Fallvignetten als Gedankenanstoss zu weiterführenden Fragen (a – d)

5. **Fallvignette 1**: Spirituelle Ressourcen

Eine Patientin hat so starke chronische Knieschmerzen, dass längere Gehstrecken nicht mehr zu bewältigen sind. Der Behandler exploriert die Folgen dieser Schmerzen für den Alltag. Dabei zeigt auch, dass diese sie daran hindern, in den nahegelegenen Wald zu gehen, um dort in Verbundenheit mit der Natur Kraft zu tanken. Der Behandler nimmt Kontakt mit dem Physiotherapeuten auf. Durch Gehtraining und Gehhilfen sind wieder längere Spaziergänge möglich.

a. Kommen Ihnen ähnliche oder vergleichbare Erfahrungen in den Sinn, in denen solche Ressourcen, die man als spirituell bezeichnen kann, von Patienten in der Behandlung wichtig wurden? Können Sie diese Situation kurz schildern? Wenn Sie keine solche Erfahrungen haben, bitten wir Sie, mit obigen Beispiel die nachfolgenden Fragen hypothetisch so zu beantworten, als hätten Sie eine solche Patientin behandelt.

b. Was war in dieser Interaktion Ihre Rolle? Was haben Sie gemacht?

c. Was hat sich in dieser Situation bewährt, was war gut?

d. Was stand in dieser Situation im Weg, was war schwierig?

6. **Fallvignette 2**: Spirituelle Belastungen

Ein Patient mit chronischen Rückenschmerzen äussert in einem Gespräch über sein Krankheitsverständnis zögerlich, dass er seine Schmerzen auch als Bestrafung durch eine höhere Macht versteht. Die Ärztin fragt nach, wofür sich der Patient denn schuldig fühle, und woher seine Vorstellung komme. Durch das Benennen der Schuldgefühle findet eine erste Entlastung davon statt.

a. Kommen Ihnen ähnliche oder vergleichbare Erfahrungen in den Sinn, in denen spirituelle Belastungen in der Behandlung wichtig wurden? Können Sie diese Situation kurz schildern? Wenn Sie keine solche Erfahrungen haben, bitten wir Sie, mit obigen Beispiel die nachfolgenden Fragen hypothetisch so zu beantworten, als hätten Sie eine solche Patientin behandelt.

b. Was war in dieser Interaktion Ihre Rolle? Was haben Sie gemacht?

c. Was hat sich in dieser Situation bewährt, was war gut?

d. Was stand in dieser Situation im Weg, was war schwierig?

7. **Fallvignette 3**: Spirituelle Aspekte in Bezug auf den Umgang im Alltag mit chronischen Schmerzen

Ein Patient äussert in einem Gespräch, dass ihn die Akzeptanz des Schmerzgefühls das Leben allgemein intensiver und bewusster leben lässt. Die Ärztin fragt nach, in welchen Situationen und Interaktionen dies besonders zum Tragen komme.

a. Kommen Ihnen ähnliche oder vergleichbare Erfahrungen in den Sinn, in denen die Sinngebung von Leiden in der Behandlung wichtig wurden? Können Sie diese Situation kurz schildern? Wenn Sie keine solche Erfahrungen haben, bitten wir Sie, mit obigen Beispiel die nachfolgenden Fragen hypothetisch so zu beantworten, als hätten Sie eine solche Patientin behandelt.

b. Was war in dieser Interaktion Ihre Rolle? Was haben Sie gemacht?

c. Was hat sich in dieser Situation bewährt, was war gut?

d. Was stand in dieser Situation im Weg, was war schwierig?

8. Haben Sie weitere Erfahrungen, Gedanken oder Anmerkungen zum Thema?
