# Supplementary material for: Developing a best practice guide for integrating spiritual care interventions in chronic pain therapy: a qualitative Delphi study
Source: Front Pain Res (Lausanne). 2025 Nov 14;6:1682702. doi: 10.3389/fpain.2025.1682702 (PMC12660185; doi:10.3389/fpain.2025.1682702)
Supplement: Supplementary file 3 [file Datasheet3.pdf]

Zweite Runde der Delphi-Befragung: Leitfaden zur Integration spiritueller Aspekte in die multimodale Schmerztherapie

Zürich, 16. Juli 2021

Sehr geehrte Damen und Herren

Anfang diesen Jahres haben Sie im Rahmen einer ersten Befragungsrunde nach Delphi Ihre klinischen Erfahrungen und Ihr Wissen im Umgang mit spirituellen Aspekten in der Behandlung von Patienten mit chronischen Schmerzen eingebracht.

Bei dieser ersten Befragungsrunde erhielten wir reichhaltige und konkrete Anregungen und Ideen, welche wir in verschlüsselter Form in einer qualitativen Inhaltsanalyse ausgewertet haben. Die Kernergebnisse dieser qualitativen Analyse haben wir in einen Gesprächsleitfaden aufbereitet. Wir freuen uns sehr, Ihnen nun diesen aus Ihrer Expertise hervorgehende Entwurf vorlegen zu können.

Im Rahmen einer zweiten Befragungsrunde, möchten wir Sie sehr gerne erneut um Ihre kritische Betrachtung und Ihre Anregungen bitten. Dazu lassen wir Ihnen **6 Fragen** zukommen. Bitte halten Sie Ihre Gedanken und Anregungen dazu wiederum schriftlich direkt im Dokument unter den entsprechenden Fragen fest. Um die Auswertung zu optimieren wurden die Zeilen des Gesprächsleitfadens im Entwurf nummeriert, so können Sie, wenn gewünscht, direkt Bezug auf einzelne Zeilen nehmen.

Wir würden uns sehr freuen, wiederum auf Ihre Mitarbeit zählen zu können. Bitte retournieren Sie dieses Dokument mit Ihren Antworten bis zum **01.09.2021** an delphi.spiritualcare@theol.uzh.ch. Für Rückfragen stehen wir gerne jederzeit zu Verfügung. Vielen Dank für Ihre Bemühungen!

Freundliche Grüsse

Das Studienteam
Prof. Dr. Simon Peng-Keller[1], Prof. Dr. Michael Rufer[2], Prof. Dr. Rahel Naef[3], cand. med. Joël Perrin[1], pract. med. Karin Hasenfratz[1]

---

[1] Professur für Spiritual Care, Universität Zürich

[2] Zentrum für Soziale Psychiatrie, Klinik für Psychiatrie, Psychotherapie und Psychosomatik, Psychiatrische Universitätsklinik Zürich

[3] Zentrum Klinische Pflegewissenschaft, Universitätsspital Zürich & Institut für Implementation Science in Health Care, Universität Zürich

FONDS NATIONAL SUISSE
SCHWEIZERISCHER NATIONALFONDS

Universität Zürich[UZH]

Smarter Health Care
National Research Programme

1. **Bildet der Gesprächsleitfaden in dieser Form Ihre Anregungen aus der ersten Runde ab? Haben Sie Ergänzungen oder Anpassungsvorschläge?**

2. **Wie schätzen Sie die Eignung des Leitfadens für die klinische Arbeit mit Personen mit chronischen Schmerzen ein (inhaltliche Kommentare sind ebenfalls sehr erwünscht)?**
   Bitte geben Sie eine Einschätzung mittels NRS 1 (= absolut unbrauchbar) – 10 (= perfekt geeignet) und führen Sie kurz schriftlich aus

3. **Wiederholt sind *sprachliche* Bilder erwähnt worden, die als hilfreich für das Gespräch erlebt werden** *(siehe: Ziele des Gesprächs > Spezifisch > Finden einer gemeinsamen Symbolsprache)*:
   a) Haben Sie gute Erfahrungen mit gewissen *sprachlichen* Bildern? Mit welchen?

   b) Würden Bildkarten/Fotos helfen? Wenn ja, in welcher Form / welche Bilder?

4. **Welche weiteren Hilfestellungen / Zusatzmaterialien könnten für solche Gespräche hilfreich sein?**

5. **Wie wichtig erscheint es Ihnen, die explorierten r/s Ressourcen und Belastungen im Patientendossier o.ä. zu dokumentieren und worauf sollte dabei geachtet werden?**

6. **Welche weiteren Hilfestellungen würden Sie für die Umsetzung des Leitfadens brauchen oder empfehlen? Welche Zusammenarbeit würden Sie empfehlen?**
