# Supplementary material for: Developing a best practice guide for integrating spiritual care interventions in chronic pain therapy: a qualitative Delphi study
Source: Front Pain Res (Lausanne). 2025 Nov 14;6:1682702. doi: 10.3389/fpain.2025.1682702 (PMC12660185; doi:10.3389/fpain.2025.1682702)
Supplement: Supplementary file 4 [file Datasheet4.pdf]

FNSNF Fonds national suisse Schweizerischer Nationalfonds
Universität Zürich[UZH]
74 NRP Smarter Health Care National Research Programme

# Leitfaden zur Integration spiritueller Aspekte in die multimodale Schmerztherapie

## 1. Rahmenbedingungen schaffen

Über religiöse und spirituelle (r/s) Aspekte zu sprechen, ist für Patienten wie auch für Ärzte, Pflegende und therapeutische Berufe oft mit Unsicherheit behaftet. Modalitäten der patientenzentrierten Kommunikation (s. Wolf A. Langewitz) sind dabei hilfreich und bilden die Grundlage der Gesprächsführung.

**Vorbereitung**: Thematik erschliessen

- Auseinandersetzung mit der eigenen r/s Haltung

**Setting**: Gesprächssetting optimieren

- Privatsphäre für persönliche Themen schaffen
- Sich bewusst dafür Zeit nehmen

**Haltung**: Offenheit signalisieren

- Bereitschaft, zwischen den Zeilen zu lesen (verbal & averbal)
- Wertungsfreies Interesse am Patienten über die Somatik hinaus zeigen

## 2. Gesprächseinstieg und Exploration

Ein Gespräch über r/s Aspekte kann über einen indirekten oder einen direkten Einstieg entstehen.

### *2.1 Indirekt*

Indirekt gelingt ein Einstieg am ehesten über das Explorieren von allgemeinen und r/s Ressourcen; über das Erkennen und Sich-Einlassen symbolischer und bildhafter Sprache der Patient*innen (s. Erhard Weiher); oder das Ergründen der individuellen Krankheitskonzepte.

**Ressourcen**

- Retrospektiv: Fragen nach bewährten intrapersonalen Bewältigungsstrategien
  - *Was hat früher geholfen, den Schmerz zu ertragen?*
  - *Was gab Ihnen jeweils „Energie“ / füllte Ihre „Batterie“?*
  - *Gab es etwas, das Ihnen als ganzer Mensch gut tat?*
- Aktuelles/zukünftiges (prospektiv): Sinnhaftigkeit des Lebens mit Erkrankung; Ziele erfragen
  - *Was gibt Ihrem Leben Bedeutung?*
  - *Beschäftigen Sie Sinnfragen?*
  - *Was gibt Ihnen Kraft?*

**Symbolsprache**

- Aufnehmen von bild- und symbolhaften Formulierungen des Patienten im Gespräch; gezieltes Erfragen innerer Bilder / Gedanken in Bezug auf den SZ
  - *Gibt es innere Bilder, die Sie in Krisensituationen als sinnstiftend / tröstend / kraftvoll / hilfreich erleben?*
  - *Helfen Ihnen positive Formulierungen und Gedanken aus schmerzfreien Phasen?*
  - *Welche Gedanken haben Sie, wenn Sie aktiv am Schmerz arbeiten (z.B. während der Physiotherapie/Ergotherapie etc.)?*
  - *Wenn Sie Ihr Befinden als Bild darstellen könnten: Wie würde dieses aussehen? Wie sah es früher aus? Wie würden Sie sich wünschen, dass es aussieht?*

**Krankheitskonzepte**

- Krankheitsverständnis, Ursachen, Ängste, Potential für Verbesserung
  - *Wie erklären Sie sich Ihre Schmerzen?*
  - *Was macht die Schmerzen erträglicher?*
- Fragen nach alternativen Therapiemethoden
  - *Für einige Menschen sind auch alternativ- oder komplementärmedizinische Methoden (wie Akupunktur, Tai-Chi, Chi-Gong Mind-Body Medicine etc.) hilfreich. Wie ist das bei Ihnen?*

### *2.2 Direkt*

Ein Gespräch über r/s Aspekte kann durch das direkte Erfragen von r/s Ressourcen oder Belastungen entstehen. Als Screening-Instrument für gesundheitsrelevante r/s Aspekte wie z.B. in einer multimodalen Behandlung eignen sich Fragebögen, z.B. der SDRQ (= Spiritual Distress and Resources Questionnaire), welche auch als Gesprächsgrundlage verwendet werden können.

**Ressourcen**

- *Für viele Menschen spielen r/s Aspekte eine Rolle im Umgang mit chronischen Schmerzen. Wie ist das bei Ihnen?*
- *Gibt es Tätigkeiten, bei denen Sie sich ganz eins mit sich selbst fühlen?*
- *Haben Sie manchmal das belebende Gefühl, Teil eines grösseren Ganzen zu sein?*

**Belastungen**

- *Fehlt es Ihnen aufgrund Ihrer Erkrankung an innerer Kraft und Inspiration?*
- *Fühlen Sie sich durch Ihre Erkrankung vom Leben abgeschnitten?*
- *Fühlen Sie sich durch Ihre Erkrankung in Ihrem Glauben oder Ihren Überzeugungen erschüttert?*

## 3. Ziele des Gesprächs

### *3.1 Allgemein*

Der niederschwellige Einbezug r/s Aspekte ist per se eine Investition in die therapeutische Beziehung und braucht nicht in jedem Fall eine spezifische, darüber hinausgehende Intervention.

Bereits das Erlauben und Anerkennen von r/s Aspekten – sofern sie eine Rolle spielen - und die Reflexion darüber zu fördern, kann etwas bewirken. Im Zentrum steht die Wahrnehmung des Menschen in seiner Ganzheit (gerade auch r/s Aspekte), darüber hinaus kommt es zu einem wichtigen Informationsgewinn über psychosoziale Hintergründe und Krankheitskonzepte.

### *3.2 Spezifisch*

Spezifische Interventionen in der multimodalen Behandlung könnten sein:

**Berücksichtigung der r/s Dimension des Patienten im gemeinsam formulierten Therapieziel**

- Förderung des Zugangs zu bestehenden r/s Ressourcen und Einbindung in therapeutische Handlungen
    - *Gezielte Ergo- und Physiotherapie, um die r/s Ressource zu ermöglichen oder erhalten (z.B. Besuch eines r/s Ortes, Körperhaltung für Meditation/Gebet, Wahrnehmen sinnstiftender sozialer Rollen, Spazierengehen in der Natur etc…)*

**Alternative / neue / unbekannte r/s Ressourcen erschliessen**

- *Was möchten Sie (wieder) tun können was ihrem Leben einen Sinn ergibt?*
- *Wie könnte man das, was man an einem aktuell nicht zugänglichen Kraftort erlebt hat, in anderer Weise aktualisieren?*
- *Was sind die sinnstiftenden Beziehungen, Werte, Aktivitäten, Erkenntnisse im Leben*

**Thematisierung und evtl. Auflösung negativer r/s (Krankheits-)Konzepte**

- Prozesshafte Begleitung und Förderung einer längerfristigen veränderten Sichtweise seitens des Patienten / der Patientin
    - *„Schmerz verunmöglicht" zu „Schmerz lehrt"*
    - *„Bestrafung" zu „Aufgabe"*
- Ermutigung und Unterstützung des Patienten zur konstruktiven Reflexion von Schuld- und Sinnfragen
- Unterstützung in der Reflexion der r/s Belastung

**Hilfreiche Sprachbilder suchen und entfalten**

- Gemeinsame nach unterstützenden Bildern für abstrakte Formulierungen suchen
    - *Baum (Thematik: Geerdet), Garten (Thematik: Zentriert), Fluss (Thematik: „im Fluss")*
    - *Sanddüne: Versuchen eine Sanddüne hochzulaufen & nicht vom Fleck zu kommen (Thematik: Kampf & Rückschläge)*
    - *Bahnhof: Am Bahngleis stehen, es kommt kein Zug (Thematik: Hoffnungslosigkeit, Planlosigkeit)*
    - *Auto: Auto, das getankt werden muss (Thematik: Ressourcen, Energiequelle)*
