# Supplementary material for: Developing a best practice guide for integrating spiritual care interventions in chronic pain therapy: a qualitative Delphi study
Source: Front Pain Res (Lausanne). 2025 Nov 14;6:1682702. doi: 10.3389/fpain.2025.1682702 (PMC12660185; doi:10.3389/fpain.2025.1682702)
Supplement: Supplementary file 5 [file Datasheet5.pdf]

FNSNF FONDS NATIONAL SUISSE SCHWEIZERISCHER NATIONALFONDS | Universität Zürich[UZH] | 74 NRP Smarter Health Care National Research Programme

Dritte Runde der Delphi-Befragung: Leitfaden zur Integration spiritueller Aspekte in die multimodale Schmerztherapie

Zürich, 04.04..2022

Sehr geehrte Damen und Herren

Endlich ist er da! Nach intensiver Auseinandersetzung mit Ihren wertvollen Kommentaren und Verbesserungsvorschlägen liegt der Leitfaden in einer neuen, präfinalen Version vor. Wir haben uns entschieden, ihn als übersichtliche Karte mit Begleitheft zu gestalten – und freuen uns sehr, Ihnen das Resultat zukommen zu lassen. Herzlichen Dank für Ihre Mitarbeit!

Gerne möchten wir Sie dazu einladen, den Leitfaden in den kommenden Wochen in Ihrem Kontext und nach Ihren Möglichkeiten einzusetzen und zu testen.

Abschliessend werden wir eine dritte Runde der Delphi-Befragung mit **4 Freitextfragen sowie 3 skalierten Frageblöcken (siehe 5.)** durchführen. Bitte lassen Sie uns Ihre Erfahrungen zur klinischen Anwendbarkeit **Ende Juni** via delphi.spiritualcare@theol.uzh.ch zukommen.

1. **Welche Erfahrungen haben Sie mit dem Leitfaden gemacht? Haben Sie Inputs zu inhaltlichen oder gestalterischen Anpassungen?**
2. **Bei (ungefähr) wie vielen Patient:innen (nominal und/oder prozentual) haben Sie in den letzten Wochen den Leitfaden oder einzelne Fragen daraus verwendet?**
3. **Wie eignet sich der Leitfaden im klin. Alltag für die Arbeit mit Personen mit chronischen SZ?**
   Bitte geben Sie eine Einschätzung mittels NRS 1 (= absolut unbrauchbar) – 10 (= perfekt geeignet)
4. **Bildet der Leitfaden in dieser Form Ihre Anregungen aus der zweiten Runde ab?**
5. **Bitte geben Sie eine Einschätzung zu Akzeptabilität, Angemessenheit und Machbarkeit der Verwendung des Leitfadens im klinischen Alltag (Fragbogen siehe Seite 3).**
   Wir haben uns entschieden, dafür validierte Fragebogen mit skalierten Antwortmöglichkeiten zu verwenden. Für die Auswertung ist es wichtig, dass Sie möglichst überall eine Antwort angeben, auch wenn die Fragen sehr ähnlich sind.

Aufgrund der logistischen Machbarkeit möchten wir Ihnen die Betaversion gerne als .pdf zum Selbstausdruck zukommen lassen. Wenn Sie zusätzlich eine gedruckte Version wünschen, nehmen Sie bitte gerne mit uns per E-Mail Kontakt auf.

Die finale Version, welche im Anschluss an diese letzte Delphi-Runde mit Ihrem Input entsteht, würden wir Ihnen sehr gerne als physisches Exemplar schicken. Dafür bräuchten wir Ihre Postadresse sowie die Anzahl der gewünschten Exemplare ebenfalls auf delphi.spiritualcare@theol.uzh.ch.

Wir möchten uns ganz herzlich für Ihre wertvolle und sehr geschätzte Mitarbeit bedanken! Wenn Sie für die weitere Verwendung des Leitfadens einen Workshop oder eine interne Weiterbildung (1/2h – 2h) durch uns wünschen, dürfen Sie gerne mit uns über karin.hasenfratz@uzh.ch in Kontakt treten.

Freundliche Grüsse

Das Studienteam

Prof. Dr. Simon Peng-Keller[1], Prof. Dr. Michael Rufer[2], Prof. Dr. Rahel Naef[3], pract. med. Joël Perrin[1], Dr. med. Karin Hasenfratz[1]

---

[1] Professur für Spiritual Care, Universität Zürich

[2] Zentrum für Soziale Psychiatrie, Klinik für Psychiatrie, Psychotherapie und Psychosomatik, Psychiatrische Universitätsklinik Zürich

[3] Zentrum Klinische Pflegewissenschaft, Universitätsspital Zürich & Institut für Implementation Science in Health Care, Universität Zürich

Skala zur Akzeptabilität der Intervention (SAI)[1]

	Stimme überhaupt nicht zu	Stimme nicht zu	Weder, noch	Stimme zu	Stimme vollkommen zu
1. Spiritual Care findet meine Zustimmung.					
2. Der Spiritual Care Leitfaden spricht mich an.					
3. Ich mag den Spiritual Care Leitfaden für chronische Schmerzpatient:innen					
4. Ich begrüße Spiritual Care bei chronischen Schmerzpatient:innen					

Angemessenheit der Intervention Skala (AIS)[1]

	Stimme überhaupt nicht zu	Stimme nicht zu	Weder, noch	Stimme zu	Stimme vollkommen zu
5. Der Spiritual Care Leitfaden scheint für chronische Schmerzpatient:innen passend zu sein.					
6. Der Spiritual Care Leitfaden scheint für den klinischen Alltag geeignet zu sein.					
7. Der Spiritual Care Leitfaden scheint im klinischen Alltag brauchbar zu sein.					
8. Der Spiritual Care Leitfaden scheint eine gute Wahl für Personen mit chronischen Schmerzen zu sein.					

Skala zur Machbarkeit der Intervention (SMI)[1]

	Stimme überhaupt nicht zu	Stimme nicht zu	Weder, noch	Stimme zu	Stimme vollkommen zu
9. Spiritual Care scheint umsetzbar zu sein.					
10. Spiritual Care scheint möglich zu sein.					
11. Gespräche zu Spiritual Care scheinen machbar zu sein.					

12. Der Spiritual Care Leitfaden scheint benutzerfreundlich zu sein.					

1. Kien, C., et al., Psychometric Testing of the German Versions of Three Implementation Outcome Measures. Global Implementation Research and Applications, 2021. **1**: p. 183–194.
