# Supplementary material for: Developing a best practice guide for integrating spiritual care interventions in chronic pain therapy: a qualitative Delphi study
Source: Front Pain Res (Lausanne). 2025 Nov 14;6:1682702. doi: 10.3389/fpain.2025.1682702 (PMC12660185; doi:10.3389/fpain.2025.1682702)
Supplement: Supplementary file 6 [file Datasheet6.pdf]

Leitfaden zur **Integration spiritueller Aspekte** in die **multimodale Schmerztherapie**

Be—gleit—heft

mit **Erläuterungen, Fragekatalog** und **Hilfsmitteln**

Schweizerischer Nationalfonds

Universität Zürich[UZH]

74 NFP Gesundheitsversorgung
Nationales Forschungsprogramm

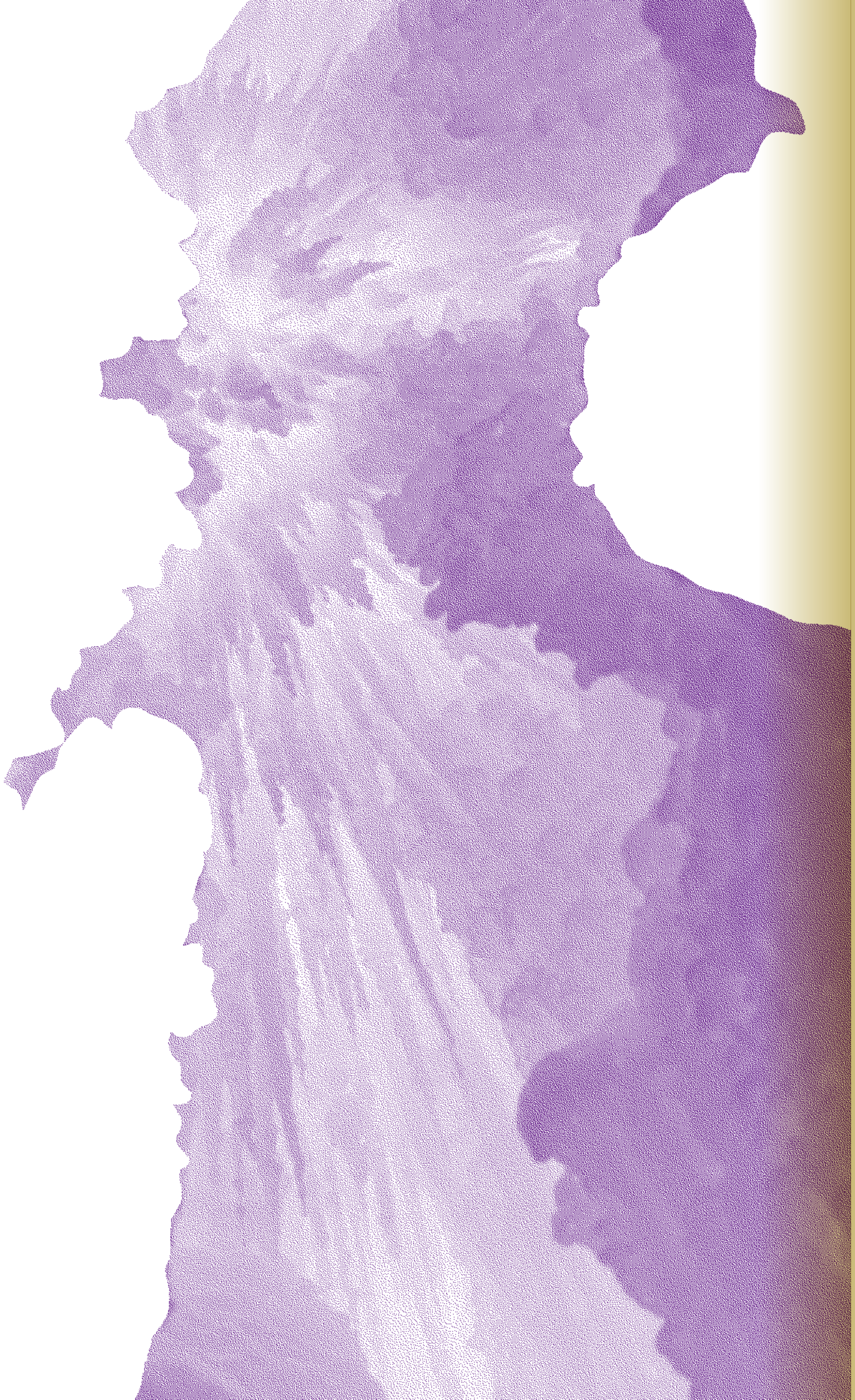

Hintergrund

Entstehung

In einer vom Schweizerischer Nationalfonds geförderten Studie zu spirituellen Ressourcen und Belastungen bei chronischen Schmerzpatient:innen gaben über 60% der befragten Patient:innen an, dass sie die Berücksichtigung spiritueller Aspekte im Behandlungsprozess wünschen[1]. Gesundheitsfachpersonen stehen diesem Wunsch grundsätzlich positiv gegenüber, sind jedoch aufgrund fehlender Modelle, knapper zeitlicher Ressourcen und wenig Erfahrung in diesem Bereich eher zurückhaltend [2,3].

Dieser Leitfaden zur Integration spiritueller Aspekte in eine multimodale Schmerztherapie wurde unter Leitung der Professur für Spiritual Care der Universität Zürich in einem mehrstufigen Prozess entwickelt. Er ist das Resultat einer Zusammenführung vorausgehender Forschungsergebnisse und einer Delphi-Studie in Zusammenarbeit mit klinischen Expert:innen verschiedener Disziplinen (Medizin, Pflege, Psychologie Physiotherapie, Ergotherapie, Sozialarbeit) und betroffenen Patient:innen. Er zeigt konkret und praxisorientiert auf, wie die spirituelle Dimension niederschwellig und patientenzentriert in eine multimodale Schmerztherapie integriert werden kann.

Der Leitfaden, der aus einer Karte und einem Begleitheft besteht, kann sowohl als praktisches, personalisierbares Hilfsmittel genutzt werden als auch als Inspiration und kleines Nachschlagewerk. Nicht alle vorgeschlagenen Fragen zu spirituellen Aspekten in dem Begleitheft sind in jedem Fall passend.

Sie verstehen sich nicht als abzuarbeitende Checkliste, sondern als Inspiration

1. **Hasenfratz K, Moergeli H, Sprott H, Ljutow A, Hefti R, Rittmayer I, et al.** Do Chronic Pain Patients Wish Spiritual Aspects to Be Integrated in Their Medical Treatment? A Cross-Sectional Study of Multiple Facilities. Frontiers in Psychiatry. 17. Juni 2021;12:685158.

2. **Rettke H, Naef R, Rufer M, Peng-Keller S.** Spiritual Care und chronischer Schmerz: Die Sicht von Fachpersonen. Eine qualitative Untersuchung. Spiritual Care. 20. Januar 2021;10(1):42–52.

3. **Perrin J, Streeck N, Naef R, Rufer M, Peng-Keller S, Rettke H.** Comparing perspectives: patients' and health care professionals' views on spiritual concerns and needs in chronic pain care - a qualitative study. BMC Health Serv Res. 26. Mai 2021;21:504.

für therapeutische Gespräche. Je nach Situation und Hintergrund werden sich gewisse Fragen dabei als hilfreich erweisen und andere weniger.

Gedacht ist dieser Leitfaden für Fachpersonen im Gesundheitsbereich wie Ärzt:innen, Pflegefachpersonen, Physio- und Ergotherapeut:innen und Sozialarbeiter:innen, die im Bereich der multimodalen Schmerztherapie arbeiten.

Die Leitidee von «Spiritualität»

Im Rahmen einer multimodalen Schmerzbehandlung bewusst von Spiritualität zu sprechen, ist sowohl für Patient:innen wie auch für Gesundheitsfachpersonen neu und kann deshalb Verunsicherungen hervorrufen. Die Vorstellungen darüber, was mit Spiritualität gemeint ist, können weit auseinandergehen. Spirituelle Aspekte sind von existentiellen und psychosozialen Aspekten nicht scharf zu unterscheiden und nicht mit religiösen Themen gleichzustellen. Sie werden oft als sehr privat wahrgenommen und nicht selten als Tabuthemen angesehen[4]. In Gesprächen mit Gesundheitsfachpersonen werden sie daher selten spontan und explizit zur Sprache gebracht. Der Leitfaden orientiert sich an der Definition von Spiritual Care der Leitlinien Spiritual Care in Palliative Care[5]:

> **«Als ‹spirituell› gelten sinnstiftende Erfahrungen, Einstellungen und Praktiken, die eine Person mit dem verbinden, was ihr Leben trägt und inspiriert. Sie können religiöser wie nicht-religiöser Art sein.»**

In dem eingangs genannten Forschungsprojekt haben sich drei klinisch besonders wichtige Aspekte herauskristallisiert: Spirituelle Ressourcen, spirituelle Belastungen (oft mit der Krankheit in Zusammenhang stehend), und spirituelle Strategien im Umgang mit chronischen Schmerzen. Um Fachpersonen ein kurzes und praxisorientiertes Screening-Instrument in die Hand zu geben, wurde ein Fragebogen zur niederschwelligen Erfassung spiritueller Ressourcen, Belastungen und Umgangsstrategien (Spiritual Distress and Resources Questionnaire, im folgenden als SDRQ abgekürzt) entwickelt und validiert[6]. Dieses kann auch vor einem entsprechenden Gespräch vom Patienten ausgefüllt werden.

Gesundheitsfachpersonen können auf unterschiedliche Weise zu einer interprofessionellen Spiritual Care beitragen. Sie übernehmen in diesem Bereich einen Grundversorgungsauftrag und ergänzen die Seelsorge als spezialisierte Form der Spiritual Care. Dieser Leitfaden beschränkt sich auf das Aufgabenfeld gesundheitsberuflicher Spiritual Care. Er soll Gesundheitsfachpersonen ein Hilfsmittel sein, um der spirituellen Dimension der Patient:innen, wo diese Behandlung wichtig sein könnte, offen und mit einem Konzept im Hinterkopf begegnen zu können. Der Einbezug spezialisierter Spiritual Care dürfte besonders in komplexen Fällen angezeigt sein.

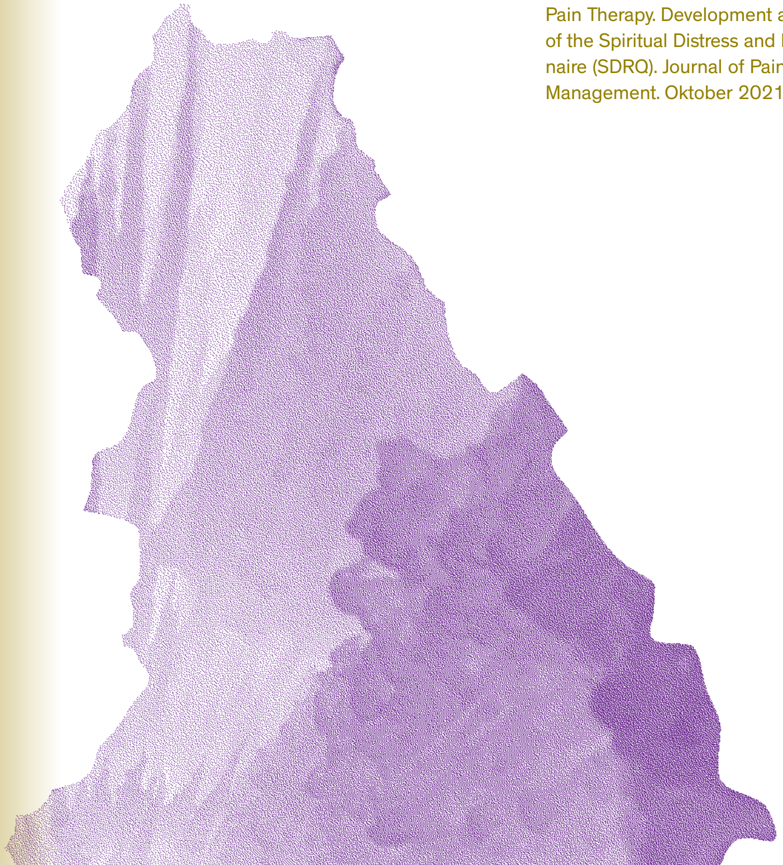

4. **Adami S, Breuning M, Bengel J, Bischoff A, Peng-Keller S.** Chronische Schmerzpatientinnen und -patienten sprechen über ihre Spiritualität. Spiritual Care. 2018;7(3):243–53.
5. **Palliative ch** (Hrsg.), Spiritual Care in Palliative Care. Leitlinien zur interprofessionellen Praxis, Bern 2018
6. **Peng-Keller S, Moergeli H, Hasenfratz K, Naef R, Rettke H, Hefti R, u. a.** Including the Spiritual Dimension in Multimodal Pain Therapy. Development and Validation of the Spiritual Distress and Resources Questionnaire (SDRQ). Journal of Pain and Symptom Management. Oktober 2021;62(4):747-756.

Leitfaden für die Gesprächsführung

Ziel

Im Zentrum steht die Wahrnehmung des Menschen in seiner Ganzheit – gerade auch mit seinen spirituellen Belangen. Da spirituelle Themen für viele durch chronische Erkrankungen belastete Menschen wichtig sind, ist es geboten, sie in Gesprächen über Krankheit, Gesundheit und Therapieziele anzusprechen. Ärzt:innen, Pflegefachpersonen, Physio- und Ergotherapeut:innen und Sozialarbeiter:innen sind oft wichtige Vertrauens- und Bezugspersonen in einer langen Krankheitsgeschichte – und deshalb auch in Bezug auf spirituelle Themen wichtige Gesprächspartner für die Patient:innen.

Gespräche oder Gesprächssequenzen über spirituelle Aspekte haben vielfältige Formen und Anlässe: Sie ergeben sich manchmal aus einer anderen Thematik heraus; es kann ein gezieltes und geplantes Gespräch über spirituelle Themen sein, beispielsweise nach einem vorausgegangenen Screening; oder es kann sich eine Gesprächsserie entwickeln, in der spirituelle Themen immer wieder aufgegriffen und vertieft werden.

Der niederschwellige Einbezug von spirituellen Aspekten ist eine Investition in die therapeutische Beziehung und braucht nicht in jedem Fall eine spezifische, darüberhinausgehende Intervention. Bereits das offene Gespräch über spirituelle Aspekte kann – sofern diese für Patient:innen wichtig sind – die Reflexion über deren Bedeutung fördern und damit als Intervention verstanden werden.
Manchmal können auch spezifische Interventionen in Bezug auf spirituelle Aspekte sinnvoll sein. Interventionsmöglichkeiten sind zum Beispiel, spirituelle Ressourcen zu erkunden und zu stärken oder in die individuellen Behandlungsziele zu integrieren, neue Ressourcen zu erschliessen oder negative Krankheitskonzepte, die zu einer spirituellen Belastung führen, anzusprechen.

Rahmenbedingungen schaffen

Gespräche mit Patient:innen über spirituelle Aspekte finden geplant oder auch spontan statt, aus einer Gesprächssituation zu anderen Themen heraus. Förderliche Rahmenbedingungen zu schaffen, ist für einen guten Gesprächsverlauf vorteilhaft.

Vorbereitung

Um spirituelle Themen offen ansprechen zu können, braucht es eine authentische und interessierte Grundhaltung diesem Themenfeld gegenüber.
Die eigene Auseinandersetzung und Reflexion darüber helfen, für die Anliegen und Weltanschauungen des Gegenübers offen und neugierig zu sein und sie von eigenen Anschauungen zu unterscheiden.

Setting

In einem stationären Setting kann es wichtig sein, eine Privatsphäre zu schaffen, um Raum für persönliche Themen zu öffnen. Ist ein Gespräch über spirituelle Themen geplant, lohnt es sich, genügend Zeit dafür zu reservieren.

Haltung

Nicht für alle Patient:innen haben spirituelle Themen gleich viel Bedeutung. Doch all jene, die darüber sprechen möchten, werden dies eher tun, wenn ihnen vom Gegenüber diesbezüglich Offenheit signalisiert wird.

Gesprächseinstieg und Exploration

Indirekter Einstieg

Ein indirekter Einstieg in ein Gespräch über spirituelle Themen ist geeignet, wenn nicht klar ist, welche Bedeutung spirituelle Aspekte für den/die Patient:in haben, und welches Vokabular er oder sie dafür benutzt.
Nicht immer führen die nachfolgend beschriebenen indirekten Zugänge zu einem spirituellen Thema, sie können auch in ganz andere, für den/die Patient:in wichtige Bereiche münden. Es kann sein, dass für den/die Patient:in spirituelle Aspekte im Moment nicht zentral sind, ihm/ihr anderes wichtiger ist, oder die Gesprächssituation dafür nicht geeignet ist. Je nach Kontext und Betreuungsprozess kann später erneut danach gefragt werden.

Im Folgenden werden mehrere Möglichkeiten beschrieben, um einen indirekten Zugang zu schaffen.

Ressourcen explorieren

Spirituelle Ressourcen können entdeckt werden über das Suchen und Explorieren von Ressourcen im generellen Sinn. Diese sind häufig eng mit anderen psychosozialen Faktoren verbunden. Sie lassen sich manchmal retrospektiv gut erkennen, manchmal eher in der aktuellen Situation.

Retrospektiv
- Was hat Ihnen früher geholfen, die Schmerzen zu ertragen?
- Wie haben Sie früher schwierige Situationen überstanden?
- Was gab Ihnen jeweils Energie/füllte Ihre «Batterie»?
- Gab es etwas, das Ihnen ganz allgemein gut getan hat?

Aktuell
- Woran halten Sie sich fest, wenn es schwierig wird?
- Gibt es etwas, das Ihr Leben mit Sinn erfüllt?
- Was macht Ihr Leben sinnvoll/lebenswert/inspirierend?
- Was gibt Ihnen Kraft?
- Wo/womit/bei wem können Sie «auftanken»? Wo können Sie aufatmen?
- Gibt es Momente, in denen Sie alle Schwierigkeiten und Schmerzen vergessen können?
- Gibt es Menschen oder Orte, die Ihnen das Gefühl der Zugehörigkeit und Verbundenheit vermitteln?

Symbolsprache und Bilder

Unsere Sprache ist voller Bilder, Metaphern, Sprichwörter und Redewendungen. Es ist für das Ansprechen spiritueller Aspekte hilfreich, genau hinzuhören, welche Begriffe, Symbole und Bilder Patient:innen ins Gespräch einbringen. Auf welche Überzeugungen, Prägungen, verinnerlichte Glaubenssätze könnten die beiläufig verwendeten Redewendungen hinweisen?

Manche Menschen können sich besser in Bildern als in abstrakten Worten mitteilen. Gelegentlich ermöglichen Bilder auch eine Kommunikation über sprachliche Barrieren und kulturelle Unterschiede hinweg. Es ist möglich, gezielt nach inneren Bildern und Metaphern zu fragen, oder diese gemeinsam mit dem/der Patient:in zu entwickeln.

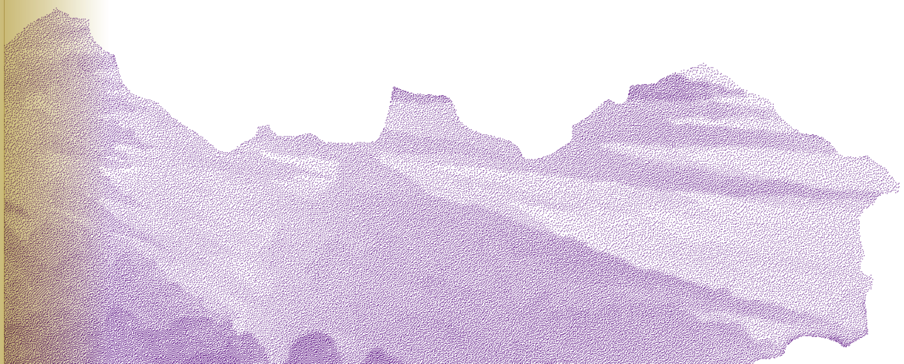

- Sie wählten gerade diese Worte (…) – was meinen Sie damit genau/was bedeutet es Ihnen?
- Wie sieht ihr Schmerz aus? Können Sie ihm einen Geruch/einen Geschmack/eine Farbe zuordnen?
- Wenn Sie Ihr Befinden als Bild darstellen könnten: Wie würde dieses aussehen? Wie sah es früher aus? Wie würden Sie sich wünschen, dass es aussieht?
- Gibt es innere Bilder, die Sie in Krisensituationen als sinnstiftend/tröstend/kraftvoll/hilfreich erleben?

Krankheitskonzepte

Es ist für einen therapeutischen Prozess wichtig zu verstehen, welches Konzept ein:e Patient:in von seiner/ihrer Krankheit oder den zugehörigen Symptomen hat: Worin sieht der/die Patient:in die Ursache seiner/ihrer Erkrankung, was sind damit verbundene Ängste? Wo und wie wird Potential für Veränderung oder Verbesserung gesehen? Manchmal kann das Krankheitskonzept auch spirituelle Überzeugungen beinhalten, auf welche in einem Gespräch eingegangen werden kann.

- Wie erklären Sie sich Ihre Schmerzen?
- Gibt es etwas, das den Schmerz erträglicher macht?
- Für einige Menschen sind auch komplementäre Methoden mit spirituellem Hintergrund (wie Tai-Chi, Chi-Gong, Mind-Body Medicine etc.) hilfreich. Wie ist das bei Ihnen?

Direkter Einstieg

Wenn es Kontext, Beziehung und vorangegangene Gespräche ermöglichen, kann direkt nach spezifischen spirituellen Ressourcen oder Belastungen gefragt werden. Auch ein systematisches Screening in Bezug auf gesundheitsrelevante spirituelle Aspekte kann einen direkten Einstieg in ein Gespräch über spirituelle Themen ermöglichen. Als schriftlicher Screening-Fragebogen kann der bereits erwähnte SDRQ-Fragebogen (= Spiritual Distress and Resources Questionnaire) dienen, welcher für einen niederschwelligen und praxisorientierten Einsatz im kulturellen Kontext der Schweiz konzipiert wurde. Die Antworten können danach als Gesprächsgrundlage verwendet werden und ebenfalls einen direkten Einstieg in ein Gespräch über spirituelle Themen ermöglichen.

Ressourcen

In einem ressourcenzentrierten Ansatz bietet es sich an, direkt nach spirituellen Ressourcen zu fragen.

- Für viele Menschen spielen Glaubensüberzeugungen/Religion/spirituelle Erfahrungen eine Rolle im Umgang mit chronischen Schmerzen. Wie ist das bei Ihnen?
- Gibt es Situationen oder Tätigkeiten, bei denen Sie sich ganz eins mit sich selbst fühlen?
- Haben Sie manchmal das Gefühl, Teil eines grösseren Ganzen zu sein? In welchen Situationen stellt sich dieses Gefühl ein?
- Fühlen Sie sich einer religiösen Gemeinschaft zugehörig?

Belastungen
Manchmal stehen spirituelle Belastungen im Vordergrund. Auch diese können direkt erfragt werden.

- Fehlt es Ihnen aufgrund Ihrer Erkrankung an innerer Kraft und Inspiration?
- Fühlen Sie sich durch Ihre Erkrankung vom Leben ausgeschlossen/abgeschnitten?
- Fühlen Sie sich durch Ihre Erkrankung in Ihrem Glauben oder Ihren Überzeugungen erschüttert?
- Wenn Sie mir das so erzählen, frage ich mich, ob Sie irgendwelche Schuldgefühle haben im Zusammenhang mit Ihrer Erkrankung? Haben Sie vielleicht manchmal das Gefühl, dass Sie etwas falsch gemacht haben?

Spezifische Interventionen

Spezifische Spiritual Care Interventionen in der multimodalen Behandlung könnten sein:

Berücksichtigung der spirituellen Dimension in gemeinsam formulierten Therapiezielen
Förderung des Zugangs zu bestehenden spirituellen Ressourcen und Einbindung in therapeutische Handlungen, z.B. durch gezielte Ergo- oder Physiotherapie, Schmerztherapie oder Alltagsplanung

- Unterstützung zum Wahrnehmen sinnstiftender sozialer Rollen, zur Pflege von tragenden Freundschaften und Beziehungen
- Spazierengehen in der Natur/Achtsamkeit
- Kreatives Schaffen ermöglichen
- Den Besuch eines wichtigen «Kraftortes» ermöglichen
- Körperliche Voraussetzungen für Meditation oder Gebet

Neue spirituelle Ressourcen erschliessen
Durch chronische Schmerzen oder Erkrankungen und die damit einhergehenden Einschränkungen ist der Zugang zu bestehenden spirituellen Ressourcen potentiell erschwert oder verunmöglicht. Es kann wichtig sein, Patient:innen zu ermutigen, sich auf die Suche nach alternativen und neuen Ressourcen zu machen und sie dabei zu unterstützen.

Dabei geht es nicht darum, dem/der Patient:in Vorschläge zu machen oder ihm/ihr gar etwas aufzudrängen. Vielmehr ist die Begleitung einer Suche nach neuen unterstützenden Praktiken, Werten und Erfahrungen gemeint. Manchmal ist es auch angebracht, zuerst einmal einen Verlust festzustellen und zu würdigen.

- Wie gehen Sie damit um, dass sie diese für Sie wertvolle/tragende Aktivität nicht mehr tun können?
- Welche anderen Aktivitäten oder Kraftorte könnten hilfreich sein?
- Was möchten Sie (wieder) tun können, was Ihrem Leben einen Sinn gibt?
- Wenn Sie Zauberkräfte hätten – was würden Sie tun?
- Welches sind die sinnstiftenden Beziehungen, Werte, Aktivitäten, Erkenntnisse in Ihrem bisherigen Leben? Wie könnten sie gefördert und aktiviert werden?
- Könnten Sie das, was Sie an einem aktuell nicht zugänglichen Kraftort erlebt haben, in anderer Weise ähnlich erleben?

Belastende (Krankheits-)Konzepte ansprechen

Die Aufklärung über die Entstehung von Schmerz und das gemeinsame Erarbeiten eines individuellen Krankheitskonzeptes ist eine wichtige Grundlage der Schmerztherapie. Wenn hierbei deutlich wird, dass spirituelle Belastungen im Zusammenhang mit der Erkrankung bestehen, kann das Ansprechen von diesen für den/die Patient:in wichtig sein. Oft ist es für ihn/sie hilfreich und entlastend, diese aussprechen und verstehen zu können.

Eine prozesshafte Begleitung und Förderung einer weniger belastenden Sichtweise kann für die Therapie von Bedeutung sein.

- Welche Bedeutung hat der Schmerz für Sie? Wenn er sprechen könnte: Was würde er Ihnen möglicherweise sagen?
- Für manche Menschen ist Schmerz nicht nur Schaden, sondern auch eine Art «Schutz». Wie erleben Sie das?
- Analogie zum Sport: Manche Menschen spielen ihr Leben mit einem «hohen Handicap». Sehen Sie Ihren Schmerz mehr als Herausforderung oder als Bestrafung?
- Gibt es Schuld- und Sinnfragen, die Ihnen im Zusammenhang mit Ihrer Erkrankung durch den Kopf gehen? Möchten Sie darüber sprechen?

Hilfreiche Sprachbilder suchen und entfalten

Die Bildsprache, die Patient:innen selbst benutzen, bietet oft einen Anknüpfungspunkt, um über spirituelle Überzeugungen und Erfahrungen zu sprechen. Gemeinsam kann nach unterstützenden Bildern gesucht werden, und diese haben das Potential, weiterentwickelt zu werden.
Es können dazu konkrete Bildressourcen verwendet werden, sprachliche Bilder gefunden werden, oder Bilder mit dem/der Patient:in gemeinsam visualisiert oder gestaltet werden – je nach Möglichkeit des spezifischen Kontextes.

Häufige Bilder für spirituelle Ressourcen

Baum – geerdet, Wachstum, Frucht bringen, im Sturm feststehen, Heimat und Schutz bieten für Vögel

Garten – zentriert, geschützt, vielfältig, immer neu und überraschend

Fluss – im Flow, Vergänglichkeit, Kontinuität, Gelassenheit

Auto – Auto, das getankt, gewartet und gesteuert werden muss, Ressourcen, Energiequelle

Bewegungsbilder – Segel, Bergsteigen, Wandern, Tanzen

Häufige Bilder für spirituelle Belastungen

Sanddüne – Versuchen eine Sanddüne hochzulaufen, nicht vom Fleck zu kommen, Kampf und Rückschläge

Bahnhof – Am Bahngleis stehen und es kommt kein Zug, Hoffnungslosigkeit, Planlosigkeit

Weiterführende Gespräche

Unabhängig davon, ob spirituelle Aspekte gezielt angesprochen und exploriert wurden oder ob ein Gespräch spontan spirituelle Themen berührt hat, kann am Ende eines Gespräches mit dem/der Patient:in geklärt werden, ob und in welchem Kontext die spirituellen Themen wieder aufgegriffen werden sollen.

- Wie möchten Sie mit dem Thema weiterfahren?
  Wann, wo und mit wem soll es wieder zur Sprache kommen?
- Ist dafür ein weiteres Gespräch angebracht?
  Oder soll das Thema nun wieder ruhen?
- Möchten Sie jemanden zusätzlich einbeziehen?
- Haben Sie Interesse an einem Gespräch mit einer Seelsorgeperson?
  Hat früher ein Kontakt bestanden? Wenn ja, besteht der Wunsch, diesen Kontakt wieder aufzunehmen?

Möglicherweise ist es auch sinnvoll, dem Patienten eine Reflexionsaufgabe mitzugeben. Dies könnte in unterschiedlichen Formen geschehen:

- Sich anhand eines Fragebogens (z.B. SDRQ) Gedanken über die eigenen spirituellen Ressourcen und Belastungen machen
- Suche nach eigenen passenden Bildern, Gegenständen, bedeutungsvoller Musik oder Ritualen
- Aufschreiben von spirituellen Ressourcen
- Eine Dankbarkeitsliste führen:
  Wofür bin ich dankbar, worauf lenke ich meinen Fokus?

Dokumentation

Die Dokumentation der Gespräche über spirituelle Themen und deren Inhalte sollte möglichst wertfrei erfolgen. Es gilt dabei einerseits – wie bei jeder anderen Dokumentation in der Krankengeschichte auch – die Privatsphäre des Patienten zu wahren und verantwortungsvoll mit den Inhalten und dem entgegengebrachten Vertrauen umzugehen. Andererseits ist eine Dokumentation wichtig für die Behandlungskontinuität und die interprofessionelle Zusammenarbeit sowie als Erinnerung für sich selbst. Sich an die Inhalte eines solchen Gespräches zu erinnern, ist nicht nur für die Fortsetzung zu einem späteren Zeitpunkt essentiell, sondern würdigt auch die Bedeutung der Thematik für den/die Patient:in.
Wenn spirituelle Aspekte (z.B. gezieltes Fördern einer spirituellen Ressource) in die allgemeinen Therapieziele einfliessen, ist es hilfreich, die Ziele festzuhalten.

Für die Dokumentationsstrukturierung können folgende Punkte berücksichtigt werden:

- Spirituelle Aspekte im Krankheitskonzept des/der Patient:in, Bilder und Metaphern
- Spirituelle Ressourcen und Belastungen (Überzeugungen, Praktiken, Erlebnisse)
- durchgeführte und geplante Interventionen
- Ziele, Abmachungen und Ansatzpunkte für weitere Gespräche

Hilfsmittel

Die vorliegende Broschüre mit Leitfaden, der SDRQ sowie weitere Hilfsmittel finden sich zum Download unter → **www.spiritualcare-leitfaden.ch**

Notizen

Impressum

Herausgeber
Karin Hasenfratz
Joël Perrin
Professur für Spiritual Care, Universität Zürich

unter Mitarbeit von
Rahel Naef
Hanspeter Mörgeli
Michael Rufer
Simon Peng-Keller

Graphische Gestaltung
Universität Zürich
MELS, SIVIC
Petra Dollinger

März 2022

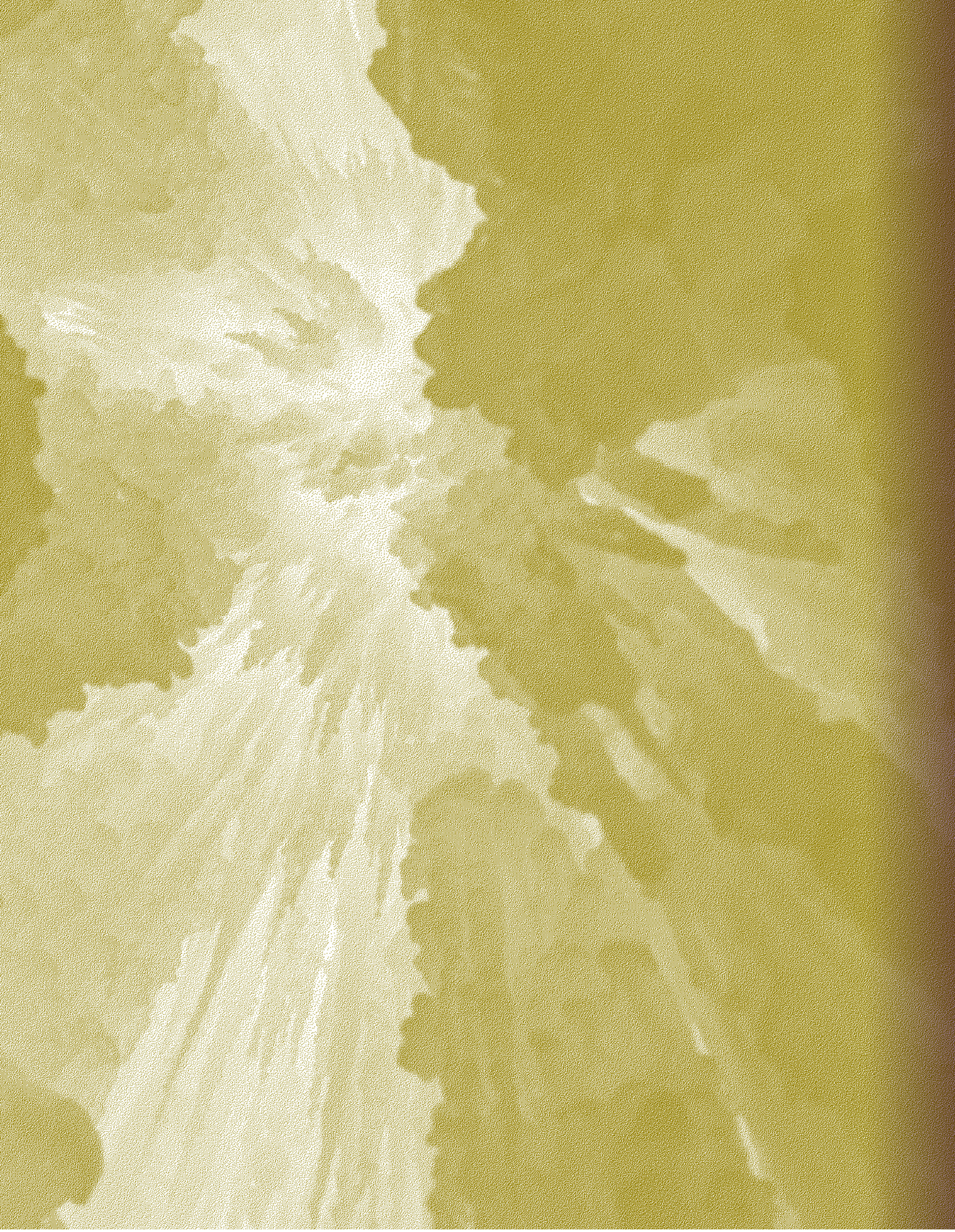

Schweizerischer Nationalfonds

Universität Zürich UZH

74 NFP Gesundheitsversorgung Nationales Forschungsprogramm
