# Supplementary material for: Developing a best practice guide for integrating spiritual care interventions in chronic pain therapy: a qualitative Delphi study
Source: Front Pain Res (Lausanne). 2025 Nov 14;6:1682702. doi: 10.3389/fpain.2025.1682702 (PMC12660185; doi:10.3389/fpain.2025.1682702)
Supplement: Supplementary file 7 [file Datasheet7.pdf]

Leit–faden

zur **Integration spiritueller Aspekte** in die **multimodale Schmerztherapie**

Ziele und Leitkonzept

Im Zentrum steht die Wahrnehmung des Menschen in seiner Ganzheit. Für viele Menschen, die unter chronischen Schmerzen leiden, sind spirituelle Themen von Bedeutung. Dieser Leitfaden soll helfen, diese Themen im Hinblick auf ihre therapeutische Bedeutsamkeit anzusprechen. Im Sinne des ressourcenzentrierten multimodalen Ansatzes liegt der Hauptfokus dieses Leitfadens auf den Ressourcen und deren Stärkung.

Als «spirituell» gelten sinnstiftende Erfahrungen, Einstellungen und Praktiken, die eine Person mit dem verbinden, was ihr Leben trägt und inspiriert. Sie können religiöser wie nicht-religiöser Art sein.

März 2022

Schweizerischer Nationalfonds
Universität Zürich[UZH]
74 NFP Gesundheitsversorgung Nationales Forschungsprogramm

Gesprächseinstieg und Exploration

Indirekt

Häufig ergibt sich die Gelegenheit, über andere Themen die spirituelle Dimension im Umgang mit der Erkrankung anzusprechen.

… über allgemeine Ressourcen (retrospektiv/aktuell)

… über das Aufnehmen von Symbolsprache, Metaphorik, Redewendungen

… über Krankheitskonzepte

Platz für eigene Notizen/Formulierungen

Direkt

Spirituelle Ressourcen

Spirituelle Belastungen

Platz für eigene Notizen/Formulierungen

Spezifische Interventionen

Der niederschwellige Einbezug von spirituellen Aspekten braucht nicht in jedem Fall eine spezifische, über das Ansprechen hinausgehende Intervention. Oft kann es genügen, vorhandene spirituelle Ressourcen wertschätzend anzuerkennen und die Reflexion darüber anzuregen.

Spezifische Interventionen innerhalb eines multimodalen Therapiekonzeptes könnten sein:

Berücksichtigung spiritueller Ressourcen in gemeinsam formulierten Therapiezielen
Mögliche neue spirituelle Ressourcen explorieren
Thematisierung und evtl. Auflösung negativer sprituelller (Krankheits-) Konzepte
Hilfreiche Sprachbilder suchen und entfalten
Platz für eigene Notizen/Formulierungen

Dokumentation

Für die Dokumentation können folgende Punkte berücksichtigt werden:

- Spirituelle Aspekte im Krankheitskonzept des/der Patient:in, genannte Bilder und Metaphern
- Spirituelle Ressourcen und Belastungen (Überzeugungen, Praktiken, Erlebnisse)
- durchgeführte und geplante Interventionen
- Ziele, Abmachungen und Ansatzpunkte für weitere Gespräche
