# Supplementary material for: Developing a best practice guide for integrating spiritual care interventions in chronic pain therapy: a qualitative Delphi study
Source: Front Pain Res (Lausanne). 2025 Nov 14;6:1682702. doi: 10.3389/fpain.2025.1682702 (PMC12660185; doi:10.3389/fpain.2025.1682702)
Supplement: Supplementary file 8 [file Datasheet8.pdf]

Fragebogen zu spirituellen Ressourcen und Belastungen

Im Umgang mit Erkrankungen können spirituelle Ressourcen und Belastungen eine wichtige Rolle spielen. Als «spirituell» gelten sinnstiftende Erfahrungen, Einstellungen und Praktiken, die eine Person mit dem verbinden, was ihr Leben trägt und inspiriert. Sie können religiöser wie nicht-religiöser Art sein.
Es kann wichtig sein, dass spirituelle Ressourcen und Belastungen in der Behandlung beachtet werden. Die folgenden Fragen sollen dabei helfen.

Der Fragebogen besteht aus drei Teilen. Kreuzen Sie bitte bei jeder Frage das für Sie zutreffende Feld an. Sie können dafür eine der beiden extremen Antworten oder ein Feld dazwischen ankreuzen. Als Beispiel die Antwort auf eine Frage nach der Schlafqualität, nachdem jemand einigermassen gut geschlafen hat:

Beispiel

	Nein, gar nicht 1	2	3	4	5	*Ja, gewiss* 6
1. Haben Sie diese Nacht gut geschlafen?	■	■	■	☒	■	■

Überlegen Sie nicht zu lange, sondern geben Sie einfach die zu Ihnen am besten passende Antwort.

Teil 1: Spirituelle Ressourcen

	Nein, gar nicht 1	2	3	4	5	*Ja, gewiss* 6
1. Gibt es Orte, wo Sie eine ganz besondere Kraft spüren?	■	■	■	■	■	■
2. Gibt es Tätigkeiten, bei denen Sie sich ganz eins mit sich selbst fühlen?	■	■	■	■	■	■
3. Gibt es Momente, in denen Sie sich selbst und die Welt um sich herum auf wohltuende Weise vergessen können?	■	■	■	■	■	■
4. Gibt es Beschäftigungen, die Sie innerlich stärken?	■	■	■	■	■	■
5. Haben Sie manchmal das Gefühl, auf eine wertvolle Weise mit einer anderen Wirklichkeit in Kontakt zu kommen?	■	■	■	■	■	■
6. Haben Sie manchmal das erhebende Gefühl, Teil eines grösseren Ganzen zu sein?	■	■	■	■	■	■
7. Gibt es für Sie bedeutsame Handlungen und Rituale, die möglicherweise zu Ihrem Glauben gehören?	■	■	■	■	■	■

Zusatzfrage

(falls Sie den obigen Fragen wenigstens teilweise zugestimmt haben)

Hindert Ihre Erkrankung Sie daran, auf die genannten spirituellen Ressourcen zurückzugreifen?	■	■	■	■	■	■

Fortsetzung auf nächster Seite …

Schweizerischer Nationalfonds
Universität Zürich UZH
74 NFP Gesundheitsversorgung Nationales Forschungsprogramm

Teil 2: Spirituelle Belastungen

Ich stimme dieser Aussage…	*...überhaupt nicht zu*					*...vollständig zu*
	1	2	3	4	5	6
1. Wegen meiner Erkrankung sehe ich keinen Sinn mehr in meinem Leben						
2. Meine Erkrankung hat meine Überzeugungen erschüttert						
3. Wegen meiner Erkrankung fehlt es mir an innerer Kraft und Inspiration						
4. Ich leide seelisch unter meiner Erkrankung						
5. Ich fühle mich mit meiner Erkrankung vom Leben abgeschnitten						
6. Meine Erkrankung hat meinen Glauben erschüttert						
7. Wegen meiner Erkrankung erscheint mir mein Leben leer						
8. Ich verzweifle an meiner Erkrankung						

Teil 3: Lebenseinstellung

Ich stimme dieser Aussage…	*...überhaupt nicht zu*					*...vollständig zu*
	1	2	3	4	5	6
1. Auch mit meiner Erkrankung habe ich ein sinnerfülltes Leben						
2. Vieles in meinem Leben fügt sich in sinnvoller Weise						
3. Auch mit meiner Erkrankung kann ich durch mein Handeln etwas Positives bewirken						
4. Ich akzeptiere, dass meine Erkrankung mein Leben beeinflusst						
5. Auch mit meiner Erkrankung gibt es etwas, das mir Halt und Zuversicht gibt						
6. Auch mit meiner Erkrankung ist mir klar, in welche Richtung mein Lebensweg gehen soll						
7. Der Umgang mit meiner Erkrankung hat mir eine tiefere Dimension des Lebens erschlossen						

Falls in diesem Fragebogen für Sie bedeutsame Themen erwähnt worden sind:	*Nein, gar nicht*					*Ja, gewiss*
	1	2	3	4	5	6
Sollten diese Themen in Ihrer Behandlung berücksichtigt werden?						

Möchten Sie zu Ihren Antworten noch etwas hinzufügen?

Schweizerischer Nationalfonds
Universität Zürich UZH
74 NFP Gesundheitsversorgung Nationales Forschungsprogramm
